# Meet changes with constancy: Defence, antagonism, recovery, and immunity roles of extracellular vesicles in confronting SARS‐CoV‐2

**DOI:** 10.1002/jev2.12288

**Published:** 2022-11-30

**Authors:** Xiaohang Chen, Huifei Li, Haoyue Song, Jie Wang, Xiaoxuan Zhang, Pengcheng Han, Xing Wang

**Affiliations:** ^1^ Shanxi Medical University School and Hospital of Stomatology Taiyuan China; ^2^ Shanxi Province Key Laboratory of Oral Diseases Prevention and New Materials Taiyuan China; ^3^ Fujian Key Laboratory of Oral Diseases, School and Hospital of Stomatology Fujian Medical University Fuzhou China; ^4^ CAS Key Laboratory of Pathogen Microbiology and Immunology Institute of Microbiology, Chinese Academy of Sciences Beijing China; ^5^ School of Medicine Zhongda Hospital, Southeast University Nanjing China

**Keywords:** ACE2, COVID‐19, engineered EVs, extracellular vesicles, nanodecoy, SARS‐CoV‐2

## Abstract

Coronavirus disease 2019 (COVID‐19), caused by severe acute respiratory syndrome coronavirus 2 (SARS‐CoV‐2), has wrought havoc on the world economy and people's daily lives. The inability to comprehensively control COVID‐19 is due to the difficulty of early and timely diagnosis, the lack of effective therapeutic drugs, and the limited effectiveness of vaccines. The body contains billions of extracellular vesicles (EVs), which have shown remarkable potential in disease diagnosis, drug development, and vaccine carriers. Recently, increasing evidence has indicated that EVs may participate or assist the body in defence, antagonism, recovery and acquired immunity against SARS‐CoV‐2. On the one hand, intercepting and decrypting the general intelligence carried in circulating EVs from COVID‐19 patients will provide an important hint for diagnosis and treatment; on the other hand, engineered EVs modified by gene editing in the laboratory will amplify the effectiveness of inhibiting infection, replication and destruction of ever‐mutating SARS‐CoV‐2, facilitating tissue repair and making a better vaccine. To comprehensively understand the interaction between EVs and SARS‐CoV‐2, providing new insights to overcome some difficulties in the diagnosis, prevention and treatment of COVID‐19, we conducted a rounded review in this area. We also explain numerous critical challenges that these tactics face before they enter the clinic, and this work will provide previous ‘meet change with constancy’ lessons for responding to future similar public health disasters.

1. Extracellular vesicles (EVs) provide a ‘meet changes with constancy’ strategy to combat SARS‐CoV‐2 that spans defence, antagonism, recovery, and acquired immunity.
2. Targets for COVID‐19 diagnosis, therapy, and prevention of progression may be found by capture of the message decoding in circulating EVs.
3. Engineered and biomimetic EVs can boost effects of the natural EVs, especially anti‐SARS‐CoV‐2, targeted repair of damaged tissue, and improvement of vaccine efficacy.

## 1 INTRODUCTION

Coronavirus disease 2019 (COVID‐19), caused by severe acute respiratory syndrome coronavirus 2 (SARS‐CoV‐2), has resulted in the illness of approximately 632 million people and the deaths of 6.6 million people worldwide, wreaking havoc on health systems (Hopkins J, 2022). The reason for the lingering haze of COVID‐19 is attributed to three main factors: (1) Difficulty in accurate and timely diagnosis: The ability to accurately screen vulnerable individuals and predict prognosis and regression are poor (Wynants et al., 2020), (2) Lack of effective therapeutic drugs: Only Molnupiravir and Paxlovid, both of which were approved by the Food and Drug Administration (FDA), have shown significant reductions in COVID‐19 hospitalization and fatality rates (Jayk Bernal et al., 2022; Tanne, 2022; U. S. Food and Drug Administration 2021a; 2021b). However, most clinical trial data for these two drugs have not been disclosed, and researchers has focused on solving production problems (Kabinger et al., 2021; Burke et al., 2022) and (3) Limited preventive efficacy: Vaccines are the most effective form of prevention, but suffer from the inability to elicit effective protection against new ‘superspread’ mutant strains such as Omicron (Garcia‐Beltran et al., 2022). Therefore, accurate and timely diagnosis, effective therapeutic drugs, and more efficacious vaccines are desperately needed. COVID‐19 occurrence and development are inextricably tied to the outcome of the fight of an organism against SARS‐CoV‐2, therefore elucidating how this ‘war’ plays out and forecasting how it can conclude is critical (Proal and VanElzakker, 2021).

**FIGURE 1:**
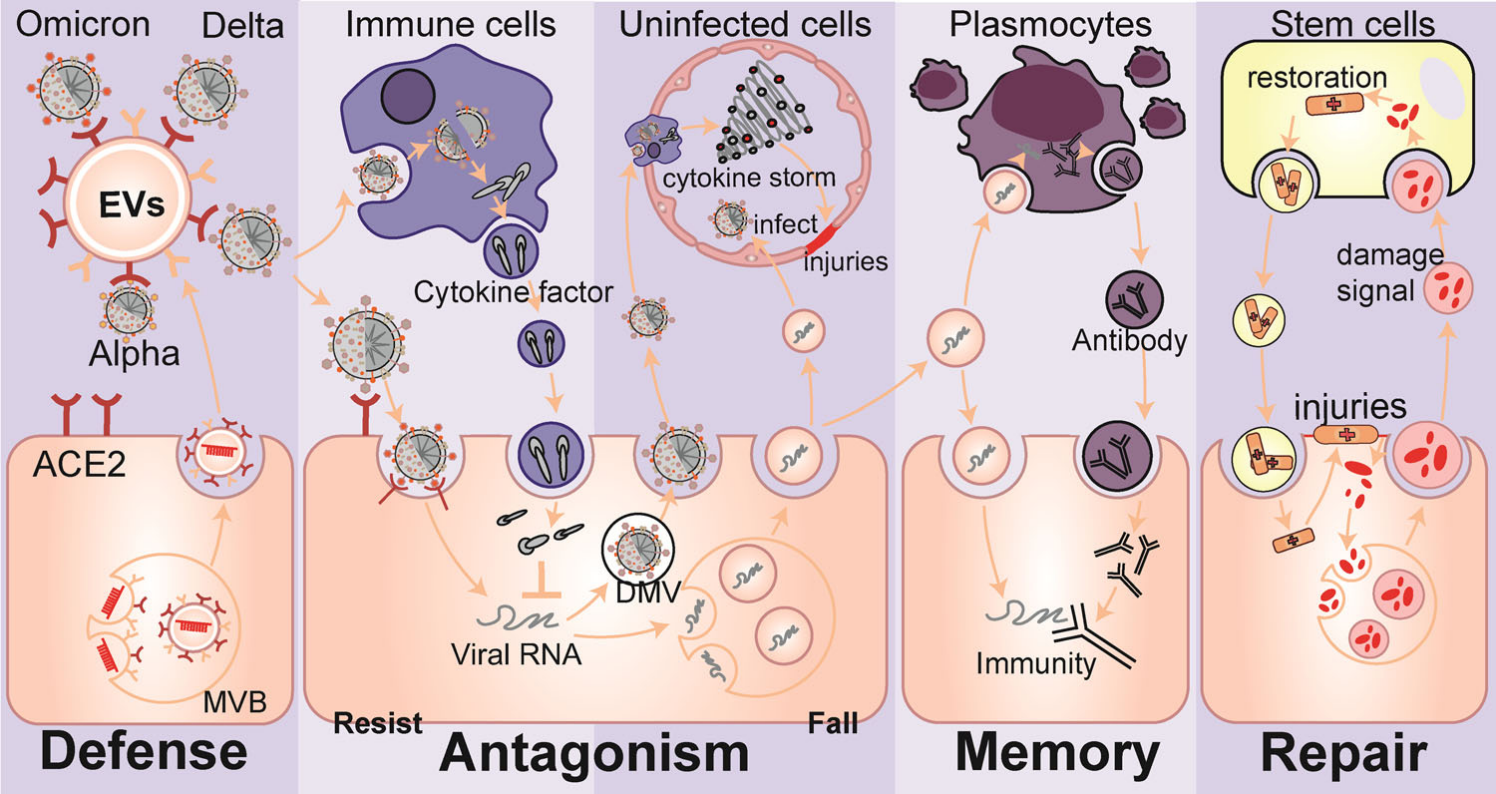
FIGURE 1 EVs may be involved in the process of the battle of the organism with SARS‐CoV‐2. (ACE2, angiotensin‐converting enzyme 2; EVs, extracellular vesicles; DMV, double‐membrane vesicles; MVB, multivesicular body.).

The body contains billions of extracellular vesicles (EVs), including exosomes and microvesicles, which are involved in nearly every physiological or pathological activity in life, particularly in confronting alien viruses (Raposo and Stoorvogel, 2013; Nolte‐’t Hoen et al., 2016). Recently, increasing evidence has indicated that during pathogenesis, the colonization of SARS‐CoV‐2 to enter cells and the subsequent replication and spread, which indirectly cause tissue damage and cytokine storms that exacerbate injuries, may all be accompanied by EVs (Figure 1). (1) When SARS‐CoV‐2 enters the cell microenvironment, EVs that carry angiotensin‐converting enzyme 2 (ACE2) as nanodecoys may prevent or assist virus colonization and entry (Berry et al., 2022; Cocozza et al., 2020; El‐Shennawy et al., 2022), (2) When infection occurs, EVs obtained by in vitro cultivation of immune cells, mesenchymal stem cells, and other cells may be used to guard against intracellular replication, spread of the virus, and act as cytokine decoys (Chutipongtanate et al., 2022; Elashiry et al., 2021; Wang et al., 2022a). Meanwhile, EVs may also become prisoners of SARS‐CoV‐2, which may play immunomodulatory effects or aid in the spread of infection (Hassanpour et al., 2020; Ning et al., 2021; Pesce et al., 2022; Tey et al., 2022), (3) EVs obtained from mesenchymal stem cells in vitro are expected to help recover from COVID‐19 related damage (Dinh et al., 2020; O'Driscoll, 2020; Sengupta et al. 2020a; Zhu et al., 2022) and (4) Circulating EVs are also potentially involved in the acquired immune response against SARS‐CoV‐2 (Barberis et al., 2021). These potential results still contain a lot of unknowns and uncertainties, but we can gain some inspiration from these processes for the diagnoses, therapy, and prevention of COVID‐19.

More importantly, EVs are cell‐derived and have outstanding biocompatibility; the lipid bilayer provides natural transmembrane ability; the protein sites on the surface allow for easy modification; and the vesicle structure allows for the loading of substances such as drug or messenger RNA (mRNA) in addition to delivering messages from their originating cells (Gurung et al., 2021; Hu et al., 2020; Meng et al., 2020; Schulz‐Siegmund , 2021). These advantages together with the defence, antagonism, recovery, and immunity roles of EVs in battling SARS‐CoV‐2, make them an excellent tool for treating, diagnosing, and preventing COVID‐19. (1) New diagnostics: Capturing circulating EVs in healthy people and patients with mild or severe COVID‐19 and decrypting the intelligence included in EVs by multi‐omics strategies to forecast major sequelae such as cytokine storms and coagulation abnormalities (Lam et al., 2021; Krishnamachary et al., 2020), (2) New therapy: the expression of ACE2 and cytokine receptors on the surface of EVs, making them a nanodecoy for SARS‐CoV‐2 and cytokines, preventing the landing of ever‐mutating SARS‐CoV‐2 and the occurrence of cytokine storms (El‐Shennawy et al., 2022; Khalaj et al., 2020; Rao et al., 2020) and (3) New vaccines: EV‐based vaccines demonstrate more effective immune protection (Jiang et al., 2022; Shen et al., 2022; Wang et al., 2022c).

Although the above studies have provided promising strategies, how to inspire ‘meet changes with constancy’ therapeutic ideas from the unique advantages of EVs in fighting SARS‐CoV‐2; how to boost the therapeutic and vaccine effects; how to utilize the intelligence interpreted from circulating EVs; and how to address the deficiency of EVs such as the limited yield, are all key issues that need to be systematically discussed and summarized. Here, we present a comprehensive review of the entire battle between SARS‐CoV‐2 and the organism in which EVs are involved, as well as the new insights that the defence, antagonism, recovery, and immunity roles of EVs provide for the diagnosis, treatment, and prevention of COVID‐19. Furthermore, engineered and biomimetic EVs boost the effect of anti‐SARS‐CoV‐2, preventing immune cytokine storms, the targeted ability to heal damaged tissues, and the efficacy of SARS‐CoV‐2‐related vaccines. Finally, we discuss several key challenges facing these strategies before entering the clinic. This work will provide new insights into the precise diagnosis, better therapy and prevention of COVID‐19, as well as useful ‘meet changes with constancy’ experiences for future possible public pandemic diseases.

## 2 DEFENCE: EVS AS A NANODECOY TO DEFEND AGAINST SARS‐COV‐2 COLONIZATION

### 2.1 EVs neutralize SARS‐CoV‐2 and guard ACE2

**FIGURE 2:**
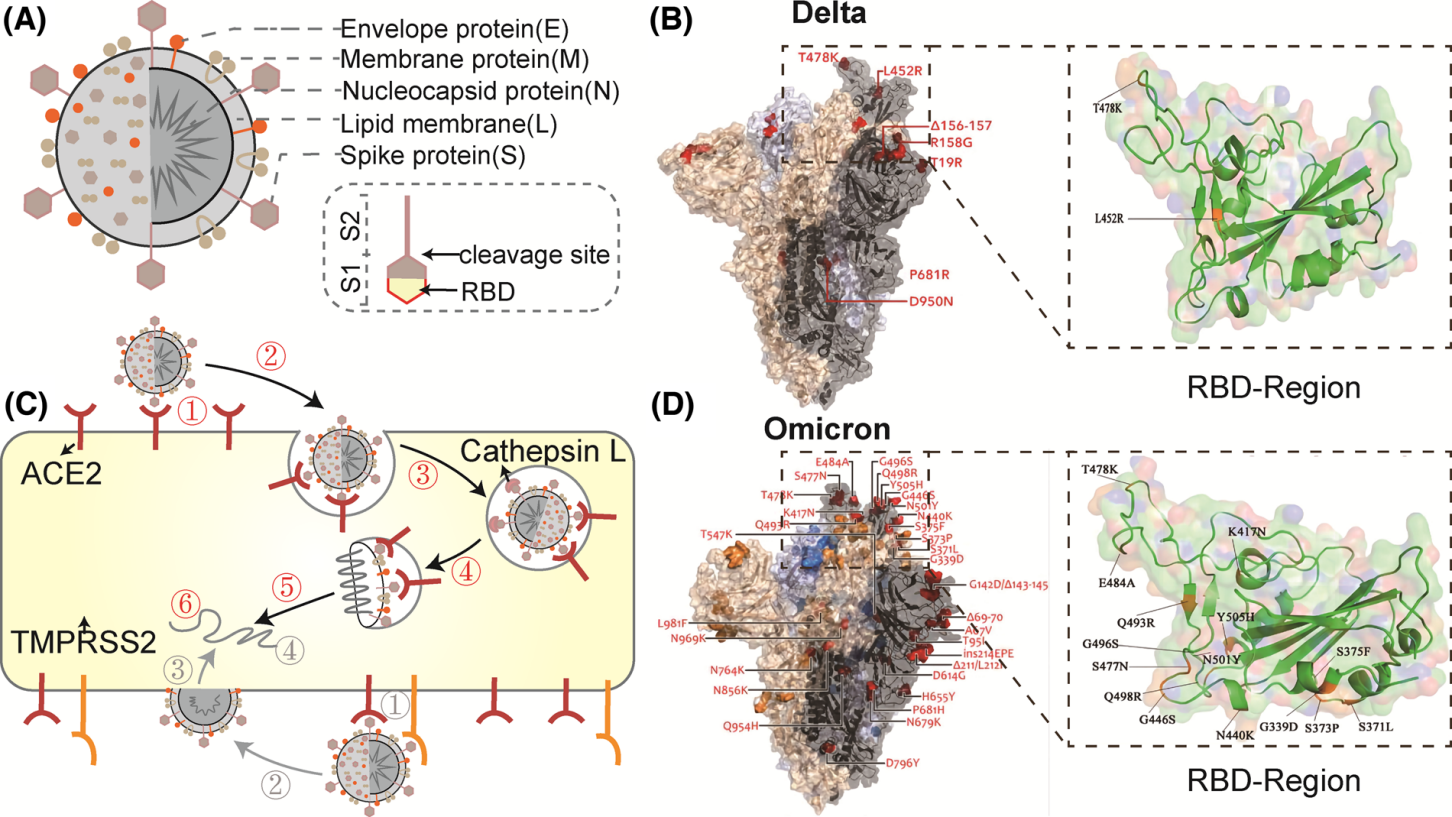
Basic information of SARS‐CoV‐2. (A) Structure of SARS‐CoV‐2. (B) Delta variant spike mutation and RBD region. (Orange color is the mutation region.) (Copyright (Kumar et al., 2022)) (C) Two mechanisms of SARS‐CoV‐2 entry into cells. Red: ①virus binding; ②internalization; ③endosomal acidification; ④cleavage of S2 by Cathepsin L; ⑤membrane fusion; ⑥uncoating of Viral RNA. Grey:①virus binding; ②cleavage of S2 by TMPRSS2; ③membrane fusion; ④uncoating of Viral RNA.(Based on copyright (Jackson et al., 2022)) (D) Omicron variant spike mutation and RBD region. (Orange colour is the mutation region.) (Copyright (Kumar et al., 2022)) (ACE2, angiotensin‐converting enzyme 2; RBD, receptor binding domain; TMPRSS2, transmembrane protease serine 2).

SARS‐CoV‐2 consists of lipid membrane (L) and four major structural proteins: nucleocapsid protein (N), membrane protein (M), envelope protein (E), and spike protein (S), containing S1 (N‐terminal domain and C‐terminal receptor binding domain, RBD) and S2 regions (Dai and Gao, 2021; Jackson et al., 2022). (Figure 2A) Before the virus enters the cell, the RBD engages ACE2 on the cell surface, and then the S protein undergoes a conformational transition with the help of proteases such as transmembrane protease serine 2 (TMPRSS2) on the plasma membrane or cathepsin L in lysosomes, in which the S1 domain is shed and the S2 domain mediates fusion with the host cell membrane and successfully enters the cell (Jackson et al., 2022). (Figure 2C) Thus, the S protein plays roles in cell entry, and mutations in SARS‐CoV‐2 are always concentrated in this region; for example, Omicron has 15 extra mutation sites (amino acid substitutions) in this region compared to Delta (Han et al. 2022). (Figure 2B, D) These mutations in the S protein usually imply an altered ability of SARS‐CoV‐2 to engage ACE2 on cells (Bowen et al., 2022). For example, Omicron has a significantly enhanced binding affinity for ACE2 compared to the original Wuhan strain virus (Kim et al., 2022c). Therefore, ACE2 exemplifies a ‘meet change with constancy’ target for COVID‐19 drug discovery in that, unlike ever‐mutating SARS‐CoV‐2 (Desai et al., 2021; Obeng et al., 2022), ACE2 in the host is unlikely to mutate.

A ngiotensin‐converting enzyme 2 is a key component of the renin‐angiotensin system (RAS), which relaxes the vascular wall and is expressed in epithelial cells of the lung, colon, kidney, heart, and blood vessels under normal physiological conditions, which is essential for the proper functioning of the cardiovascular system, the inflammatory homeostasis of the body, and tissue health (Gheblawi et al., 2020; Trougakos et al., 2021). In addition to occupying ACE2 on the cell surface, intracellular replication of SARS‐CoV‐2 causes a decrease in ACE2 expression, which eventually leads to ACE2 catabolism to ACE, and when ACE2/ACE signalling is blocked, then severe reactions may occur in the body, such as vasoconstriction, hypokalaemia, acute respiratory distress syndrome, and acute renal failure (Beyerstedt et al., 2021; Bourgonje et al., 2020). Therefore, the protection of ACE2 is crucial for improving the prognosis of the disease and reducing mortality. An interesting topic is how to protect ACE2 from physiological dysfunction while not allowing SARS‐CoV‐2 to land and how to protect against infection. Researchers have developed many strategies, such as using soluble ACE2 as a cheat for SARS‐CoV‐2, but they face the problems of easy degradation (soluble ACE2 lasting only a few hours) and low bioavailability (Wysocki et al., 2021). To address this problem, we previously created hACE2‐T27F‐R273Q, a mutant R273Q that inactivates the enzymatic activity of hACE2, and then attempted to alter the important hydrophobic or charged motif of ACE2—the T27F site—which will enable a higher affinity for SARS‐CoV‐2‐RBD. In vivo, hACE2‐T27F‐R273Q has a half‐life of up to 33 h, which is three times longer than hACE2, and neutralizes RBD with a 2–7 times higher efficiency than hACE2 (Zheng et al., 2022). However, these effects are far from ideal.

Surprisingly, smart cells use ACE2‐containing EVs as bait to neutralize SARS‐CoV‐2 and prevent its colonization of the cell membrane, thereby blocking virus colonization and entry into the cell as well as guarding ACE2 (Cocozza et al., 2020; El‐Shennawy et al., 2022; Keller et al., 2020). This process was tracked in real time by Lim et al. using high‐speed atomic force microscopy (HS‐AFM) (Lim et al., 2021). A variety of cell‐secreted EVs include ACE2, which acts as an effective bait to neutralize the RBD region of the S1 protein in SARS‐CoV‐2, greatly reducing the infectivity of SARS‐CoV‐2 to cells (Cocozza et al., 2020). The amount of ACE2 expression on the membrane surface of EVs is closely related to the cellular origin as well as the signal transduction of the microenvironment (Xie et al., 2021). For instance, the high levels of interferon (IFN)‐1 and IFN‐2 in the microenvironment help cells to enhance the expression of ACE2 (Smith et al., 2020; Ziegler et al., 2020), which may also potentially enhance ACE2 abundance in EVs. In vitro experiments confirmed that IFN‐α/β treatment increases ACE2 expression on the surface of EVs and enhances the attraction to SARS‐CoV‐2 (Zhang et al., 2021b). When exposed to SARS‐CoV‐2, a subpopulation of EVs called defensosomes is also activated, and upon entry into the cell, the intracellular autophagy‐related protein 16‐1 (ATG16L1) senses the signal and increases the expression of ACE2 on the surface of EVs, which then binds and prevents virus entry, and purified defensosomes could be used as a new antiviral strategy (Ching et al., 2022).

**FIGURE 3:**
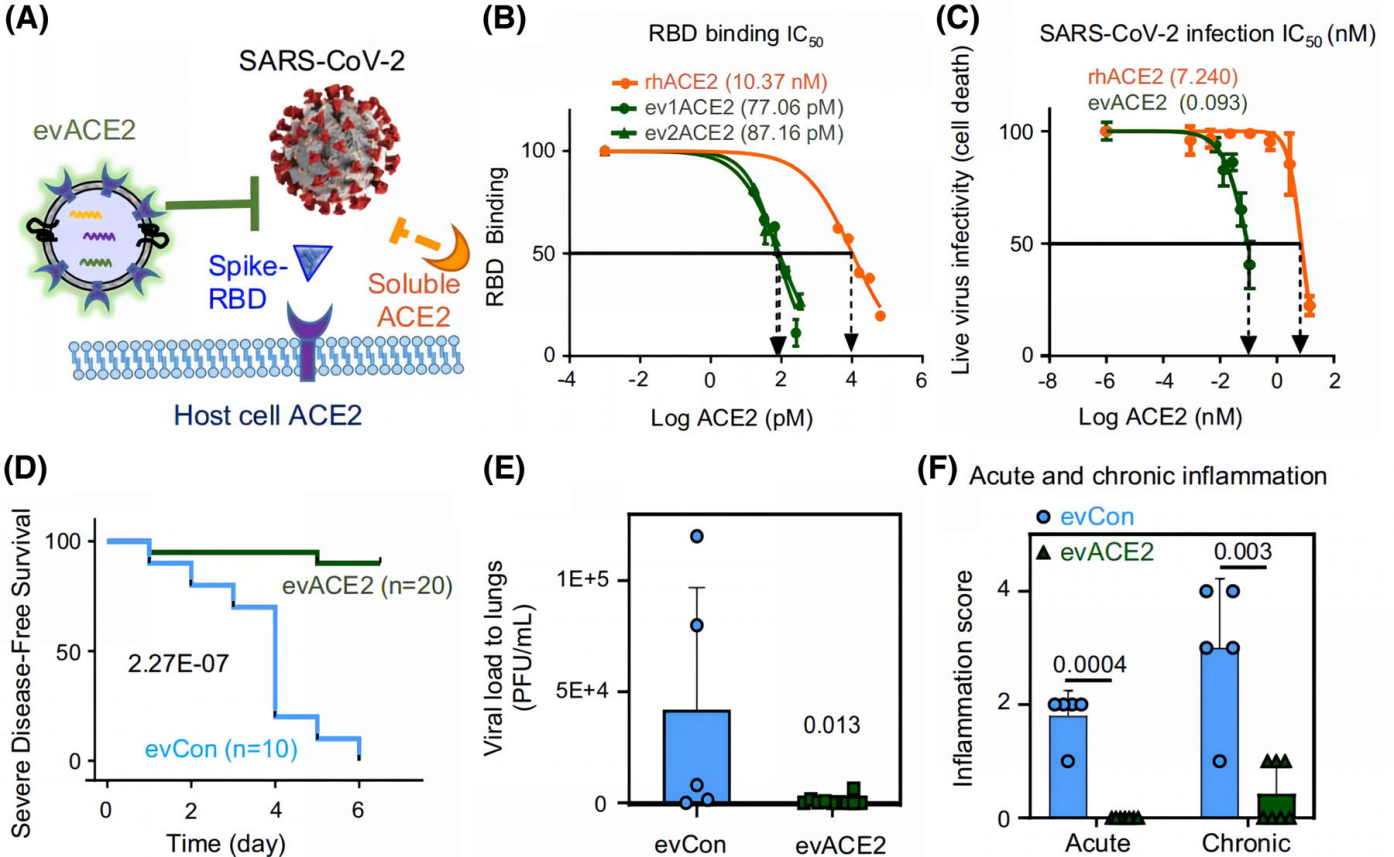
Compared to soluble recombinant human ACE2 (rhACE2), EV‐derived ACE2 (evACE2) has a stronger ability to neutralize SARS‐CoV‐2, reduce inflammation, and increase the survival rate. (A) Schematic diagram of evACE2 and soluble ACE2 competitively binding to SARS‐CoV‐2 with host cell ACE2. (B) The half inhibition amount (IC50%) of rhACE2 (orange), evACE2 (ev1ACE2 from HEK293T cells and ev2ACE2 from HeLa cells) (green) binding 16 nM RBD. (C) Half‐inhibitory concentrations (nM) of live virus infectivity in ev1ACE2 (green line) and rhACE2 (orange line) during wild‐type SARS‐CoV‐2 infection. (D) Survival rates of SARS‐CoV‐2(10,000 pfu)‐infected K18‐hACE2 disease‐free mice severely after receiving intranasal of evCon (blue) and 130 μg evACE2 (green). (E) Pulmonary viral load, and (F) acute and chronic inflammation scores of SARS‐CoV‐2(10,000 pfu)‐infected K18‐hACE2 mice after intranasal evCon (blue) and 130 μg evACE2 (green) administration at Day 5/6 post infection. (Copyright (El‐Shennawy et al., 2022)) (ACE2, angiotensin‐converting enzyme 2; Con, control; ev, extracellular vesicles; HEK293T, human embryonic kidney 293; RBD, receptor‐binding structural domain).

Additionally, compared to soluble recombinant hACE2, EV‐derived ACE2 has a stronger ability to neutralize SARS‐CoV‐2 (El‐Shennawy et al., 2022). El‐Shennawy et al. found that circulating EVs expressing ACE2 in the plasma of COVID‐19 patients could compete with ACE2 on the cell surface to neutralize SARS‐CoV‐2 (Figure 3A). Circulating EVs expressing ACE2 exhibited a 135‐fold greater ability to bind SARS‐CoV‐2‐RBD and a 60‐80‐fold greater ability to inhibit SARS‐CoV‐2 infection than recombinant hACE2 alone (El‐Shennawy et al., 2022). (Figure 3B, C) EVs with high ACE2 expression could act as decoys for SARS‐CoV‐2 in the organism, preventing normal viral colonization and RAS system damage caused by ACE2 abnormalities and reducing the risk of major complications and death. (Figure 3D‐F) Therefore, ACE2‐rich EVs are expected to be a new strategy for defence against SARS‐CoV‐2. Inspired by the natural defence capacity of EVs, many researchers have provided solutions for maximizing neutralization capacity and protecting ACE2 by engineered EVs. (Table 1)

**TABLE 1:** Engineered EVs for combating COVID‐19

EVs type (source)	Isolation; modification; storage	Administration method; dosing interval	Effects	Refs.
EVs (Fibroblasts)	Concentrated 100×; transfection (beta‐catenin and transcription factor GATA‐4).	Not mentioned.	In vitro: improve survival of SAR‐CoV‐2‐infected cells may be due to the inhibition of mTOR signalling activation by such engineered EVs.	Ibrahim et al., 2022
Exosomes (Expi293™ cells)	ExoQuick‐CG (SBI, System Biosciences); Transient transfection; −80 °C (long‐term)	Given escalating doses, 10^8^–10^10^, of EXO‐CD24 by inhalation, QD, for 5 days.	In vitro: inhibit PMA‐induced cytokine/chemokine secretion. In vivo: reduce cytokine release and lung inflammatory reactions and increase survival. Preclinical experiments: effective in reducing inflammatory markers and cytokines/chemokines.	Shapira et al., 2022
Exosomes (HEK293T)	Ultracentrifuge; transfection(hACE2); pDA‐modified.	Transtracheal‐injected; PDA@Exosome: 2.5 mg/kg.	In vivo: significant reduction in the level of inflammation. In vitro: EVs‐hACE2 bind SARS‐CoV‐2 competitively with cells and have significant anti‐inflammatory and antioxidant effects.	Ma et al., 2022
NVs (HEK293T)	Ultracentrifuge; transfection (hACE2); HA‐modified; lyophilized under sucrose protection.	Inhalation; three times/day interval 4 h for 1 days.	In vivo: NVs–HA–sucrose reduce the pseudovirus bioluminescence signal in the lungs of hACE2 overexpressing transgenic mice. In vitro: IC_50: D614G variant (16.3 μg/ml); wild‐type pseudo‐virus (9.5 μg/ml).	Zhang et al., 2021a, 2021b
NVs (LSCs)	Ultracentrifuge; concentrated 100 ×; 4 °C (1 w) or −80 °C (long‐term).	Intra‐nasal and intratracheal; 10^10^ particles/kg on days 2, 3, 4 and 5 postinfection.	In vivo: lower immunological reaction, lung fibrosis and the virus's expression level in cynomolgus macaques. In vitro: bind and neutralize spike S1 and SARS‐CoV‐2 mimics.	Li et al., 2021
Exosomes (LSCs)	Ultracentrifuge; LSCs; co‐incubation; lyophilized (trehalose and DPBS re‐suspended).	Inhalation; 10^10^ particles per kg; two doses, one week apart.	In vivo: induce systemic immunity, such as lung RBD specific IgG antibody, and mucosal immune sIgA antibody, and activate Th1‐associated CD8+ and CD4+ T‐cells; faster removal of SARS‐CoV‐2 simulators from lungs; fight severe lung inflammation caused by live virus infection. In vitro: enhance the uptake of antigen ‐ presenting cells.	Wang et al., 2022c
Exosomes (Lung‐Secretome and HEK‐Secretome)	100‐kDa amicon centrifugal filter unit (Millipore Sigma, Burlington, MA, USA); lyophilized (trehalose and DPBS re‐suspended); #electroporation.	Jet nebulization; 10^9^ particles per kg of body weight; single dose.	In vivo: distribute in both the upper and lower respiratory tracts of the lung, penetrate the mucus layer of the respiratory tract, effectively induce IgG and sIgA production and clear pseudoviruses.	Popowski et al., 2022
NVs#(HEK293T &THP‐1)	Immunomagnetic beads; HEK293T, transfection ACE2; sonicated for mixing.	Inhalation.	In vitro: inhibit SARS‐CoV‐2 infection, neutralize and reduce inflammatory factors (IL‐6 and GM‐CSF). In vivo: reduce acute lung inflammation in mice.	Rao et al., 2020
NVs#(HEK293T#& RAW264.7)	Ultracentrifuge; HEK293T, transfection ACE2; RAW264.7 cells, treated by LPS and TNF‐α; sonicated for mixing; NVs were incubated with HAMA microspheres for 1 h at a ratio of 2:1 (w/w).	Aerosol inhalation.	In vitro: neutralize SARS‐CoV‐2 and immune factors such as IL‐6, IL‐1β, TNF‐α. In vivo: reduce the rate of infection and alleviate the inflammatory response during the hyperinflammatory period of COVID‐19, significantly prolonging the residence time of NVs in the respiratory tract.	Wang et al., 2022b
EVs; NVs(HEK293FT)	High‐speed centrifugation or ultracentrifugation; transfection (ACE2 or Mut‐sACE2)	Not mentioned.	In vitro: inhibit a variety of virus strains mutations, even SARS‐CoV‐2 mutant strains that are resistant to mAb antibodies and sACE2 inhibitors.	Gunnels et al., 2022
EVs (HEK293T)	Tangential flow filtration (TFF) (300 kD); transfection (CD9ΔTM4‐ sACE2).	Intraperitoneal injection. 1 times/day for 6 days. (200 μg/mice).	In vitro: protect against WT, variants of S‐containing pseudovirus and authentic WT and Delta variant.#In vivo: reduced the rate of infection and inflammatory response in K18‐hACE2 mice.	Kim et al., 2022a
EVs (HEK293T)	Ultracentrifuge; transfection (ACE2).	Intranasal.	In vitro: blockage against virus that enters the cells.#In vivo: mice S‐pseudovirus positive rates (non‐treatment: EVs‐Control groups: EVs‐ACE2 group = 16.2%: 13.1%: 0.13%).	Wu et al., 2022
EVs (HEK293T)	Ultracentrifuge; Transfection (CD63, embedded anti‐SARS‐CoV‐2 nanobody); ‐20°C.	Not mentioned.	In vitro: bind SARS‐CoV‐2 S protein.	Scott et al., 2022
EVs (Vero‐E6)	Ultracentrifuge; transfection (PM‐ACE2).	Tail vein‐injected; (100 μg/mice) at day^‐1^.	In vitro: PM‐ACE2‐EVs’ neutralizing effect are 10 times greater than that of ACE2‐EVs. In vivo: effectively neutralize SARS‐CoV‐2 and reduce the viral load in hACE2 mice.	Xie et al., 2021
EVs (HEK293T)	Differential ultra‐centrifugation; transfection (SARS‐CoV‐2 S protein RBD‐tagged).	Injected intravenously; 150 μg/mice.	In vitro: SARS‐CoV‐2 S protein RBD‐tagged EVs target ACE2‐expressing cells.#In vivo: SARS‐CoV‐2 S protein RBD‐tagged EVs target tissues with high ACE2 expression and reduce SARS‐CoV‐2 infection.	Fu and Xiong, 2021
EVs (HEK293T)	Differential ultra‐centrifugation; HEK293T cells transfection (CD63‐VHH72).	Not mentioned.	In vitro: CD63‐VHH72‐EVs can bind SARS‐CoV‐2‐RBD and neutralize SARS‐CoV‐2, dose‐dependently reducing infection.	Scott et al., 2022
ELNs (Ginger)	Ultracentrifuge.	Intratracheal, 5×10^8^/kg.	In vitro: GELNs miRNA inhibit SARS‐CoV‐2 and Nsp12 and prevent SRAS‐CoV‐2 cytopathic effect (CPE). In vivo: GELNs aly‐miR396a‐5p inhibit the lung inflammation induced by Nsp12.	Teng et al., 2021
Exosomes (HEK293T)	SEC; pre‐mixing of mRNAs ((i)LSNME; (ii) a functional form of spike) with polycationic lipids.	Injected; the LSNME/S^W1^ EVs vaccine was administered on day 1, 21 and 42.	In vivo: activation of CD4^+^ and CD8^+^ T‐cell immunity followed by the production of antibodies to S and N proteins with stable antibody levels over time in mice.	Tsai et al., 2021
Exosomes (red blood cells)	Differential ultra‐centrifugation; incubate;	Vaccinated subcutaneously three times on days 0, 7, and 21.	In vivo: elicit robust epitope specific CD8^+^ T cell response.	Shen et al., 2022
EVs (HEK293T)	Transfection (S proteins of SARS‐CoV‐2).	Injected; 2* DNA^s‐EV^ and 1*S‐EV or 2* DNA^s‐EV^ and 1*S‐Trim or 3* S‐EV were administered on day 1, 21 and 42.	In vivo: the combination of DNA^s‐EV^ and DNA^S‐Trim^ and S‐EV produced strong cellular immunity and a significant reduction in neutralizing antibody titres in mice.	Polak et al., 2020
OMVs (S. typhimurium)	Ultracentrifuge; Co‐incubation: RBD‐Spy‐His; PBS/15% glycerol.	Inhalation on day 1, 21 and 42.	In vitro: S protein effectively capture RBD‐OMV.#In vivo: the RBD‐OMV vaccine avoid weight loss due to viral infection, enhance antibody titres, significantly lower viral titres, and reduce lung inflammation.	Jiang et al., 2022

Abbreviations: ACE2, angiotensin‐converting enzyme 2; CD, Cluster of differentiation; COVID‐19, the coronavirus disease 2019; E, envelope protein; ELNs, exosome‐like nanoparticles; EVs, Extracellular vesicles; GELNs, Ginger‐derived ELNs; GM‐CSF, granulocyte‐macrophage colony‐stimulating factor; HA, hyaluronic acid; hACE2, human ACE2; HAMA, methacrylate hyaluronic acid;HEK293T, human embryonic kidney 293; IL, interleukin; L, lipid membrane; LPS, lipopolysaccharide; LSCs, lung spherocytes; M, membrane protein; N, nucleocapsid protein; Nsp12, RNA‐rich polymerase; NVs, nanovesicles; OMV, outer membrane vesicles; pDA, polydopamine; PM‐ACE2, palmitoylation‐ACE2; RBD, Receptor‐binding structural domain; S, spike protein; SEC, size exclusion chromatography; THP‐1, Human leukemia monocytic cell; TNF, tumour necrosis factor; w/w, weight by weight.

### 2.2 Engineered EVs amplify the therapeutic efficacy in neutralizing SARS‐CoV‐2

#### 2.2.1 Engineered EVs

Many researchers confirmed that enhancing ACE2 expression on EVs by gene editing in the source cells is a viable strategy. EVs harvested from cells transfected with ACE2 is the simplest way to promote their neutralization of SARS‐CoV‐2 (Wu et al., 2022). By gene editing, it is possible to promote not only the expression of ACE2, but also the biogenesis of EVs. Xie et al. found that human cells pack ACE2 into EVs through S‐palmitoylation of two key residues (Cys141 and Cys498). When cells were overexpressed zinc‐finger DHHC‐type palmitoyltransferase 3 (ZDHHC3), the amount of ACE2 in EVs was significantly increased, as was the yield of EVs. Thus, the palmitoylation of cells can be manipulated to produce modified EVs with large amounts of ACE2 on their surface, and these EVs exhibit high efficacy and potency against SARS‐CoV‐2 in vitro and in vivo (Xie et al., 2021).

**FIGURE 4:**
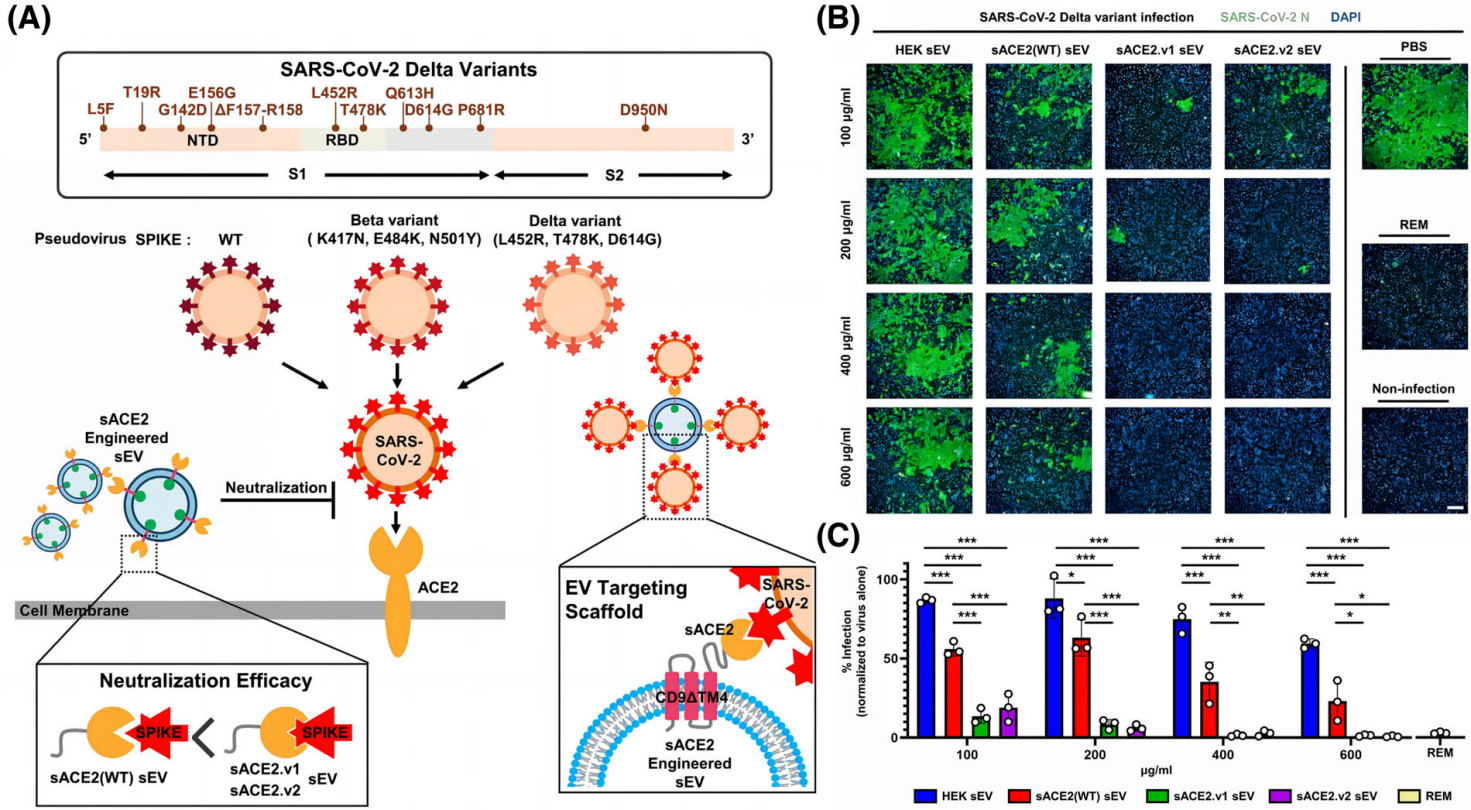
Strategy of CD9ΔTM4 on EVs as a scaffold for sACE2 to neutralize SARS‐CoV‐2. (A) Schematic diagram of CD9ΔTM4 on sEV as a scaffold for wild‐type (WT) sACE2 and mutant sACE2 (sACE2.v1 and sACE2.v2) to neutralize pseudovirus, wild‐type (WT), Delta, and Beta variants. (B) Immunofluorescence staining of Vero E6 cells infected with the SARS‐CoV‐2 Delta mutant strain and treated with different concentrations of HEK293T cell‐derived sEV, sACE2 (WT) sEV, sACE2.v1 sEV, and sACE2.v2 sEV. Positive control: Remdesivir (REM). (The nuclear diamidino‐2‐phenylindole (DAPI) of Vero E6 cells is blue, and SARS‐CoV‐2 N protein is green.) (Copyright (Kim et al., 2022a)) (ACE2, angiotensin‐converting enzyme 2; CD, Cluster of differentiation 9; EV, Extracellular vesicles; HEK293T, human embryonic kidney 293).

The surface of EVs is rich in tetraspanin, including cluster of differentiation 9 (CD9) and CD63, which can be used as scaffolds to insert different protein fragments (Jankovičová et al., 2020). Several researchers have found that increasing the expression of cell surface CD9 can also promote the production of EVs. These researchers used CD9 (with transmembrane region 4 deleted) as a scaffold, attached mutant sACE2 (with higher affinity for binding SARS‐CoV‐2), and transfected this scaffold sequence into cells, which generated EVs containing the affluent sACE2, with a high affinity for SARS‐CoV‐2 S protein, which were effective against different mutant strains (Kim et al., 2022a). (Figure 4A, B) Scott et al. suggested that overexpression of ACE2 on the surface of EVs to act as a nano‐bait is not as effective as constructing EVs that can directly block the activity of SARS‐CoV‐2 (Scott et al., 2022). The single‐stranded recombinant variable region (VHH72) was shown to directly bind the RBD region of β‐coronavirus (Yu et al., 2020). Inspired by this feature, the researchers constructed a complex inserting VHH72 on the Ex2.4 site (the optimal outer loop and location for embedding targeting proteins) of CD63, and transfected it into human embryonic kidney 293 (HEK293T) cells. These engineered EVs could directly engage the RBD region of SARS‐CoV‐2 and neutralize pseudotyped SARS‐CoV‐2. Compared to CD63‐EVs, CD63‐VHH72‐EVs dose‐dependently reduced SARS‐CoV‐2 infection (Scott et al., 2022). However, that the RBD is a mutation‐prone region (Shah et al., 2020a), and this strategy is not conducive to dealing with the constantly mutating SARS‐CoV‐2.

#### 2.2.2 Biomimetic EVs

Focusing on the advantages of EV transmembrane ability and biocompatibility, but with the difficulties of low yield and content heterogeneity, many scholars have turned to focus on cell membranes (Ilahibaks et al., 2019; Herrmann et al., 2021; Willms et al., 2018). They found that artificial cell membrane‐based nanovesicles (NVs), formed by evacuating the cell contents and extruding the cell membrane, not only retain the advantages of EVs, especially possessing receptors such as ACE2, but also overcome some of the above difficulties (Rao et al., 2020).

HEK293T cell‐derived NVs that transfected with ACE2, which contained 140 pg of ACE2 per 1 μg NV, had a potent neutralizing effect on SARS‐CoV‐2 and significantly reduced the symptoms of acute lung injury in vivo (Rao et al., 2020). Moreover, scholars found that lung spherocytes (LSCs), a mixture of mesenchymal cells and resident lung epithelial cells (both type I and type II pneumocytes), are most enriched in expressing ACE2, with approximately 2.1 × 10^6^ receptors per cell. Li et al. created LSCs‐NVs as SARS‐CoV‐2 nanodecoys and found that SARS‐CoV‐2 adsorbed on LSC‐NVs was rapidly endocytosed by macrophages and cleared. Monkey infection models showed that LSC‐NVs were still visible in the lungs after 72 h. Four consecutive doses of LSC‐NVs decreased the immune response of the organism, and reduced lung fibrosis (Li et al., 2021). Furthermore, researchers have attempted to increase the residency time of biomimetic NVs in the lungs to improve their bioavailability, and modifying NVs with hyaluronic acid is a feasible option (Zhang et al., 2021a).

Encouragingly, Gunnels and his colleagues comprehensively evaluated mutant ACE2 and wild‐type ACE2 transfected HEK293FT cell‐derived NVs and EVs obtained by different centrifugation methods and discovered that they both inhibited SARS‐CoV‐2 pseudovirus in an ACE2 dose‐dependent manner, with no significant differences in NV and EV efficacy. In addition, mutant SARS‐CoV‐2 strains that were resistant to mAb antibodies and sACE2 inhibitors, as well as Delta and Lambda, were all effectively inhibited by this nanodecoy strategy. This study shows that the antiviral effects of a nanodecoy containing ACE2 may be unaffected by the type of EVs or the purification technique used, and that, given the benefits, NVs could be a good substitute (Gunnels et al., 2022).

Notably, although the strategy mentioned above has shown some positive results, EVs as a ‘weapon’ for virus defence still leave numerous issues to be considered: (1) Comprehensive blocking strategy (Obeng et al., 2022): EV nanodecoy therapy, which is one of the block approaches of entry for SARS‐CoV‐2, is expected to block cells with high ACE2 expression (Wang et al., 2021a). However, certain immune cell types such as macrophages can use endocytosis rather than ACE2‐dependent methods to take up the SARS‐CoV‐2 virus (Wang et al., 2021a). Thus, a comprehensive blocking strategy should be considered, (2) Indications: A serious disease will develop only in a minority of patients when viral invasion has already taken place. How can candidates for such treatment be identified? Van der Made CI et al. reported that loss‐of‐function variants of X‐chromosomal toll like receptor 7 (TLR7) can lead to severe infections caused by SARS‐CoV‐2 (Van der Made CI et al., 2020), which means that EV nanodecoy therapy should be combined with other methods to identify candidates for serious disease. Various entirely distinct results, such as those listed in the next section, also present a challenge to these strategies.

## 3 ANTAGONISM: EVS ANTAGONISM WITH SARS‐COV‐2 INSPIRES DIAGNOSIS AND THERAPY

### 3.1 Antagonism between EVs and SARS‐CoV‐2

#### 3.1.1 EVs as accomplices in promoting SARS‐CoV‐2 infection

The potential use of EVs as nanodecoys for defencing SARS‐CoV‐2 and the efficiency of this strategy have both been demonstrated by several in vivo animal experiments (El‐Shennawy et al., 2022; Xie et al., 2021). However, it is unclear what the fate of the ACE2‐containing EVs nanodecoys that bind SARS‐CoV‐2 is. Recent studies have found some evidence that EVs bound to SARS‐CoV‐2 may promote SARS‐CoV‐2 infection of cells. Tey et al. discovered that EVs derived from HEK293T cells enriched with ACE2 could dose‐dependently promote SARS‐CoV‐2 infection of cells, which may be related to the easier uptake of EVs by cells (Tey et al., 2022). When the degradation of EVs and the channel of EVs are uptake by cells was blocked with bafilomycin A1 (BafA1) (which neutralizes lysosomal acidification) or cytochalasin D (a cytoskeleton inhibitor), this facilitation from ACE2‐rich EVs was significantly reduced, but the precise mechanism is unknown. This study used live viruses as opposed to the pseudoviruses used in many earlier studies, and it applied different effector cell and EV concentrations. A more open and experimental design for replication and comparability in studies of this field is also presented as a challenge due to the different results.

Berry et al. also discovered evidence that EVs originating from the mucus of human nasal epithelial cells (mu‐sEV) increased SARS‐CoV‐2 infection of cells (Berry et al., 2022). They demonstrated that SARS‐CoV‐2 exists in both ‘closed’ or ‘open’ states and that, following SARS‐CoV‐2′s binding to ACE2 on mu‐sEV, the activated protease TMPRSS2 on mu‐sEV cleaves the S1/S2 boundary, exposing the structural domain of SARS‐CoV‐2 bound to the cell surface and leaving the S protein in an ‘open’ conformational state prior to fusion. This assisted action may depend mainly on the activation of the protease TMPRSS2, whereas no activation of the TMPRSS2 was detected in the study by Tey et al. These in vitro studies indicated that, depending on their surface proteins, EVs of various cellular origins exert diverse effects in response to SARS‐CoV‐2. There is not enough proof to show how this process in the organism actually works. But the above results have shown some challenges for using exogenous EVs as SARS‐CoV‐2 nanodecoys, in terms of whether the focus should be on the source of EV or whether switches need to be added to their surface proteins to prevent cell uptake.

#### 3.1.2 EVs as a therapeutic agent against SARS‐CoV‐2

##### EVs from immune cells

Once the first line of defence of the cell fails, SARS‐CoV‐2 will successfully enter the cell and begin to engage in a fierce battle with that cell (Florindo et al., 2020; Shah et al., 2020b). When the body is highly immune, the cells will promptly release EVs with abnormal messages, and recruit surrounding intrinsic immune cells such as dendritic cells and macrophages (Robbins and Morelli, 2014). The cells generate a local immune response by releasing inflammatory cytokines and chemokines, attracting T lymphocytes and monocytes to stop the spread of the virus (Ahmed‐Hassan et al., 2020). Immune cell‐derived EVs, such as macrophages‐derived EVs, are enriched with receptors including interleukin‐6 (IL‐6) and granulocyte‐macrophage colony‐stimulating factor (GM‐CSF) on their surface and can likewise act as nanodecoys to neutralize abnormal immune inflammatory factors (Bonaventura et al., 2020). Wang et al. confirmed the anti‐cytokine storms and anti‐SARS‐CoV‐2 effect of immune cells using primary peritoneal M2 macrophage‐derived EVs and found that the effect was much stronger than the effect of immobilized cell lines including RAW264.7 possibly because of the combined effect of their microRNA (miRNA) and protein interactions, reducing TNF‐α by approximately 78% and IL‐α by approximately 65% in plasma while also significantly reducing the level of ROS production and multiorgan damage caused by oxidative stress (Wang et al., 2022b). In addition, therapeutic dendritic cell‐derived EVs were also found to accumulate in the lung after intravenous injection, and their contents, transforming growth factor beta‐1 (TGF‐β1) and TGF‐β1R complexes, could act as immunomodulators to reduce ACE2 expression on the surface of lung epithelial cells and prevent viral landing in vitro (Elashiry et al., 2021).

##### EVs from mesenchymal stem cells

Mesenchymal stem cell (MSC)‐derived EVs were shown to inhibit SARS‐CoV‐2 replication in a dose‐dependent manner because the contents of EVs act as a direct inhibitor of SARS‐CoV‐2 replication, and EV interactions with lung epithelial cells interfere with hormone‐related immune signalling that reduces infectious viral particle production and release (Chutipongtanate et al., 2022; Park et al., 2021). Seventeen miRNAs have been discovered in MSC‐EVs that may directly target the 3′ untranslated region (UTR) of SARS‐CoV‐2, a highly conserved region, and that five of them, including miR‐181a‐5p, can bind to SARS‐CoV‐2 and block its activity (Park et al., 2021). Most the five miRNA binding sites that they discovered are conserved, but no studies have been performed to explore why 3′ UTR of SARS‐CoV‐2 is conserved. This strategy of locating and inhibiting SARS‐CoV‐2′s conserved region may be another example of ‘meet changes with constancy’.

Neural stem cell (NSC)‐derived EVs also show innate antiviral activity, due to the presence of large amounts of P‐element‐induced weak testis interacting RNA (piRNA) (Yu et al., 2020). PiRNA is a small noncoding RNA that silences genes and has immune‐like functions during virus invasion (Adashev et al., 2021). For example, mouse NSCs can release piRNA‐containing exosomes/microvesicles (Ex/MVs) to provide intrinsic immunoprotection against SARS‐CoV‐2 infection. The antiviral piRNA of human NSC‐derived Ex/MVs was boosted more than twofold after exposure to various RNA fragments of SARS‐CoV‐2. Conversely, the antiviral effects were significantly reduced when piRNA was impaired. These results suggested that piRNA in Ex/MVs is an efficient strategy for silencing SARS‐CoV‐2 (Ikhlas et al., 2022).

#### 3.1.3 EVs as ‘prisoners’ of SARS‐CoV‐2

##### EVs hijacked by SARS‐CoV‐2

**FIGURE 5:**
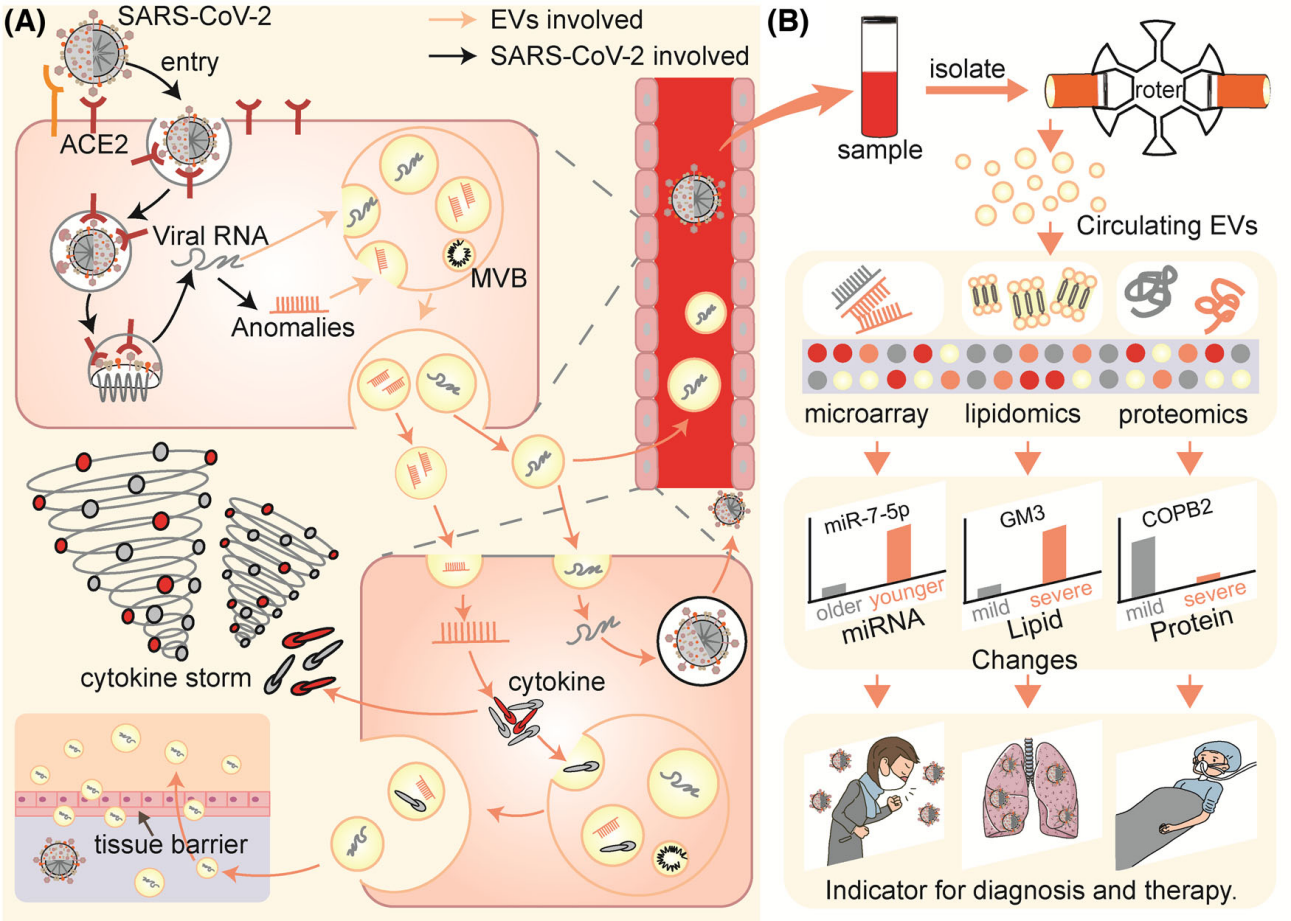
The possible process of EVs becoming ‘prisoners’ of SARS‐CoV‐2, and the circulating EVs changing for therapy hint and diagnosis. (A) EVs are hijacked by SARS‐CoV‐2 for distant organ infection and cytokine storms. (B) Multiomics sequencing of circulating EVs reveals its’ changes and provides another window for diagnosis. (ACE2, angiotensin‐converting enzyme 2; MVB, multivesicular body; GM3, ganglioside monosialylate dihexose; COPB2, coatomer protein complex subunit beta 2).

When the immunity of the organism is low and the defence line falls, the virus replicates and multiplies in large numbers within the cell and not only forms viruses through virus‐induced double‐membrane vesicles (DMVs), but these viral RNAs will be carried by encapsulation in EVs (Wong et al., 2021) due to EV biogenesis being similar to DMV in that budding, sorting, and shedding to generate distinct vesicle structures, as well as uptake (Wolff et al., 2020). Pironti et al. critically discussed the potential outcomes of EVs transporting SARS‐CoV‐2 fragments or viral fragments that might stimulate the immune system for their antigen priming activity, while the whole virion hidden in larger vesicles could potentially elicit viral dissemination (Pironti et al., 2021). Therefore, SARS‐CoV‐2 may begin a new round of relentless destruction by taking the ‘Bus’, namely, EVs. EVs are ‘pass’ communicators to all parts of the organism and may be forced to carry SARS‐CoV‐2 viral fragments for further distant spread as the ‘prisoners’ (Elrashdy et al., 2020; Elrashdy et al., 2021; Pesce et al., 2022; Pironti et al., 2021) (Figure 5A). For example, SARS‐CoV‐2 RNA fragments can be detected in EVs (Ning et al., 2021), and EVs containing S2 or S1 proteins are more readily taken up by ACE2‐expressing cells (Verta et al., 2022). SARS‐CoV‐2 spike‐derived fragments are present in the exosomes of COVID‐19 patients, but are particularly enriched in mild COVID‐19 patients, and these exosomes can be used as a source of antigen presentation to enhance immune responses and through promote T‐cell activation (Pesce et al., 2022), whereas exosomes from severe COVID‐19 patients may lead to further deterioration of the COVID‐19 illness. Exosomes containing SARS‐CoV‐2 spike‐derived fragments from COVID‐19 patients have not been specifically discussed in terms of how they induce different immune responses. It is important to emphasize that there is a lack of direct evidence on whether the SARS‐CoV‐2 fragment carried by EVs is infectious.

##### Occurrence of cytokine storm

After EVs are hijacked by SARS‐CoV‐2, immune cells release large amounts of EVs containing inflammatory factors when they receive an excessive amount of abnormal information (Mazini et al., 2021). Various cells, including lung epithelial cells, followed and released EVs, which encouraged inflammation and then were trapped in a vicious cycle. The application of an EV inhibitor (GW4869) was shown to significantly alleviate asthma and lipopolysaccharide (LPS)‐induced inflammatory responses in pneumonia and reduce the production of proinflammatory factors (Kulshreshtha et al., 2013). This information suggests that EVs are involved in and responsible for the occurrence of cytokine storms, which cause severe tissue damage and are the main cause of death in COVID‐19 patients (Xia et al., 2021). Therefore, if these EVs can be intercepted, it will bring new hope for the prevention, diagnosis, and treatment of COVID‐19.

### 3.2 Intercept circulating EVs for inspiring COVID‐19 diagnoses, therapy and prevention

After SARS‐CoV‐2 enters cells and infects lung tissues, the immunity and physiological state of the body and the pathogenicity of SARS‐CoV‐2 will forecast the outcome of this battle (Deng and Angelova, 2021). During the cellular struggle with SARS‐CoV‐2, both the content and surface composition of EVs change (Cappellano et al., 2021). Due to the important physiopathological role of EVs in vivo and the affluent content they encapsulate, they may reflect more information about the organism. Under the protection of EVs, RNA and proteins are more easily preserved than proteins extracted directly from blood (Yáñez‐Mó et al., 2015). Thus, deciphering their information is expected to be another window into the body. Using proteomics, lipidomics, and RNA sequencing, plasma circulating EVs from COVID‐19 patients were found to show significant changes in proteins, lipids, and miRNAs (Lam et al., 2021). These findings may provide useful information for diagnoses, provide hints for therapy, and predict disease prognosis. (Figure 5B) (Table 2)

**TABLE 2:** Changes of circulating EVs in COVID‐19 patients

Samples	EVs isolation	Method	Change indicators	Ref.
76 (26 healthy people and 50 patients)	Invitrogen total exosome isolation kit.	Lipidomics; metabolomics.	GM3	Song et al., 2020
78 COVID‐19 patients	Differential centrifugation; polymer precipitation.	Lipidomics; steroidomics; proteomics.	RAFT lipid metabolism, cholesterol, IL‐6, and caspase3.	Lam et al., 2021
84 COVID‐19 patients	Ultracentrifuge.	Proteomics.	TF, EN‐RAGE, IL‐18R1, TGFβ‐1, ST2, TRAIL‐R2, IFN‐γ, IL‐6RA, NPX, and FIt3L.	Krishnamachary et al., 2021
24(7 healthy people and 17 patients)	Exo‐Spin Exosome Plasma Purification Kit.	Shotgun proteomics.	CRP, A1AG1, A1AG2, CXCL7, SAMP and ZAZG, CCD34, and C4BPA.	Barberis et al., 2021
261 (60 selected patients and 201 unselected consecutive enrolled patients)	bead‐based EV‐capture and flow cyto­metric analysis by MACSPlex human Exosome Kit (Miltenyi).	CytoFLEX flow cytometer.	CD142.	Burrello et al., 2022
67 participants (34 positives and 33 negatives) and 16 healthy people	Ultracentrifuge.	H uman Tissue Factor Activity Assay.	CD142.	Balbi et al., 2021
100 patients with COVID‐19 with moderate and severe disease and 28 healthy people	Centrifuge.	ELISA	TF (tissue factor).	Rosell et al., 2021
58 (12 healthy people and 46 patients)	ExoQuick.	ELISA.	SARS‐CoV‐2 S1 and N protein, CIII‐10, SNPH, NCLX, MCU, Humanin, MOTS‐C, CI‐6, CD81, Myo‐6, VDAC1, LETM1, NMDAR1, and TSOP	Peluso et al., 2022
Case report	Not mentioned.	Not mentioned.	SARS‐CoV‐2 S protein, and HLA‐II.	Goodlet et al., 2021
41(10 healthy people and 31 patients)	Immunoprecipitation.	LC‐MS.	COPB2.	Fujita et al., 2021
28(8 healthy people and 20 patients)	ME kit (ME‐020P‐Kit).	Proteomics.	TnC, Fgβ, TNF‐α, IL‐6, and CCL5.	Sur et al., 2021
61(31 COVID‐19 patients and 30 health people)	Total Exosome Precipitation Reagent from Invitrogen (Carlsbad, CA, USA).	Luminex instrument (Invitrogen, Waltham, MA, USA)	Neutrophil elastase (NE).	Lascano et al., 2022
30(13 mild and 17 mild to severe patients)	AnTibody of CHoice and Enzymatic Release (EV‐CATCHER) assay.	Small RNA sequencing.	hsa‐miR‐146a and hsa‐miR‐126‐3p.	Mitchell et al., 2021
53 patients	Ultracentrifuge.	P roteomics.	TNF superfamily and IL‐6 family members, TF, t‐PA, vWF and MB, PRSS8, REN, HGF, CD163, RAGE, and caspase3/7.	Krishnamachary et al., 2020
31(10 healthy people and 21 patients)	Ultracentrifuge.	CytoFLEX S flow cytometer.	CD235a, CD14, CD8, CD19, CD4, and CD146.	Kudryavtsev et al., 2021

Abbreviations: A1AG1, Alpha‐1‐Acid Glycoprotein 1; C; C4BPA, complement component 4 binding protein alpha; CCL5, C‐C motif chemokine ligand 5;CD, cluster of differentiation; CI‐6, subunit 6 of NADH‐ubiquinone oxidoreductase (respiratory chain complex I); CIII‐10, subunit 10 of cytochrome b‐c1 oxidase (respiratory chain complex III); COPB2, coatomer protein complex subunit beta 2; CRP, C‐reactive protein; CXCL7, C‐X‐C Motif Chemokine Ligand 7; EN‐RAGE, extracellular newly identified receptor for advanced glycation end products binding protein; Fgβ, fibrinogen beta; GM3, ganglioside monosialyl dihexosyl; HGF, Hepatocyte Growth Factor; HLA‐II, human leukocyte antigen‐II; IFN, infereon; IL‐6, interleukin 6; IL‐6RA, IL‐6 receptor subunit alpha.; LETM1, leucine zipper EF‐hand containing transmembrane 1 protein; MB, Myoglobin; MCU, mitochondrial calcium uniporter protein; MOTS‐C, mitochondrial open‐reading frame of the 12S rRNA‐c; Myo‐6, myosin VI; NCLX, mitochondrial Na+/Ca++ exchanger; NMDAR1, N‐methyl‐D‐aspartate receptor 1; NPX, normalized protein expression; PRSS8, Serine Protease 8; RAGE, renal tumour antigen; REN, Renin; SA‐MP, San Andreas Multiplayer; SNPH, syntaphilin; ST2, IL‐1RL1; TF, tissue factor; TGF, tumour growth factor; TnC, Tenascin‐C; TNF, tumour necrosis factor; t‐PA, tissue fibrinogen activator; TRAIL‐R2, TNF‐related apoptosis‐inducing ligand receptor 2; TSOP, translocator protein; VDAC1, voltage‐dependent anion‐selective channel protein 1; vWF, von Willebrand factor.Severe acute respiratory syndrome coronavirus 2.

#### 3.2.1 Improve the sensitivity of SARS‐CoV‐2 detection

Reverse‐transcriptase quantitative polymerase chain reaction (RT‐qPCR) is the current gold standard for the detection of SARS‐CoV‐2 RNA, but there are cases of missed detection because the current main sampling site is the upper respiratory tract, using sampling media such as pharyngeal swabs and nasal swabs (Kucirka et al., 2020). The main receptor of SARS‐CoV‐2, ACE2, is not only highly expressed in the respiratory tract, but also present in various parts of the cardiovascular and gastrointestinal tracts (Ni et al., 2020). Based on this presence, circulating plasma testing may be an adjunct, but studies have shown that circulating plasma testing has a low overall sensitivity (< 41%) (Azghandi and Kerachian, 2020). By contrast, circulating EVs that assist SARS‐CoV‐2 infection may also protect the RNA from degradation (Cappellano et al., 2021), which is expected to be a strategy to reduce missed detection rates.

Ning et al. used a CD81 antibody to collect EVs in plasma, fused the captured EVs with liposomes carrying reagents such as reverse transcriptase and measured them using an enzyme‐linked immunosorbent assay‐type test (Ning et al., 2021). A salient example involved in improving the sensitivity of SARS‐CoV‐2 detection is that this method confirmed SARS‐CoV‐2 infection in six individuals with lung images compatible with COVID‐19 patients but negative nasal swab diagnosis diagnosed these individuals as early as the first day after infection. Furthermore, even if patients confirm a negative nasal swab diagnosis that matches the discharge criterion, residual SARS‐CoV‐2 may still be detected by this method, implying that SARS‐CoV‐2 has not been fully left. However, this method is currently unable to detect active infection SARS‐CoV‐2 RNA indicators, indicating that more work is needed. Although the active infection is still in its early stages, this type of research has the potential to provide guidance on the auxiliary diagnosis of complete cure versus likely recurrence in COVID‐19 patients (Tao et al., 2021). In addition, some advantages of the original PCR testing may be weakened if this approach can be adopted. For example, since the upper respiratory tract is the colony for the Omicron variant (Piersiala et al., 2022), circulating EVs may not accurately reflect the real situation. If the sampling can be applied to extend to other biological fluids of the upper respiratory tract, the possibilities of its application will increase.

#### 3.2.2 Predict the onset of symptoms

As the virus mutates, the emergence of Omicron represents a highly infectious, but less pathogenic strain of the virus, while the proportion of asymptomatic infections increases (Nyberg et al., 2022; Garrett et al., 2021). Thus, there is still a need to focus on when patients may become symptomatic or experience progression. In COVID‐19 patients, SARS‐CoV‐2 hijacks the lipid metabolism of the host, providing an energy source for its reproduction and forming an infection‐prone milieu (Abu‐Farha et al., 2020; Pei et al., 2021). Using high coverage lipidomics and steroidomics, Lam et al. found dysregulated RAFT lipid metabolism of EVs in COVID‐19 patients, with altered extracellular localization of progerin‐1 (Lam et al., 2021). In addition, altered cholesterol homeostasis is one of the key host pathways mediating SARS‐CoV‐2 infection of cells. Cholesterol levels were significantly dysregulated in EVs from COVID‐19 patients, with reduced cholesterol levels in mild COVID‐19 patients and significantly elevated levels during periods of hyperinflammation.

Many glycosphingolipids in circulating EVs are also altered, including the ganglioside monosialylate dihexose (GM3), which is the only pathologically altered lipid that strongly and negatively correlates with CD4^+^ T‐cell numbers (Song et al., 2020). As the disease progresses, GM3 accumulates in the circulating EVs of the patient and suppresses the activation of CD4^+^ T cells. The decrease in lymphocytes will lead immune dysregulation and viral rampage (Guo et al., 2021). Thus, these shifting lipid messages indicate that viruses have settled into the host and are ready to replicate and spread for manifesting symptoms. If this information is detected in a timely manner, the virus will be removed from the ‘cellular cradle’ much sooner, preventing future transmission. For example, the abnormal fluctuation of levels of protein, lipid, and miRNA in circulating EVs of COVID‐19 patients can be monitored through dynamic tracking. Many new breakthroughs have been made in the use of sensing technologies that capture and decode information in EVs for disease detection (Li et al., 2022), providing new ideas for timely monitoring of the conditions of patients with COVID‐19. Obviously, several experiments and clinical studies should be performed to validate the feasibility of new technologies.

#### 3.2.3 Forecast the brewing cytokine storm

When SARS‐CoV‐2 infects cells, the immune system of the body goes into overdrive and the number of inflammatory components in EVs may become one of the indications to predict whether COVID‐19 will result in a poor prognosis. Plasma EVs containing the IL‐6 and tumour necrosis factor (TNF) superfamilies, as well as caspase‐3, are greatly increased in severe COVID‐19 patients, indicating that the body enters a highly inflammatory state (Krishnamachary et al., 2020). Higher levels of tenascin‐C (TNC) and fibrinogen‐β in plasma EVs of COVID‐19 patients may trigger proinflammatory cytokines via the nuclear factor‐κB (NF‐κB) pathway, leading to increased levels of TNF‐α, IL‐6, and C‐C motif chemokine ligand 5 (CCL5) (Sur et al., 2021). The extracellular newly identified receptor for advanced glycation end‐products (EN‐RAGE) protein, an inflammatory marker known to be closely associated with infection‐related death, was also found to be substantially elevated in EVs from COVID‐19 patients who died (Krishnamachary et al., 2021).

**FIGURE 6:**
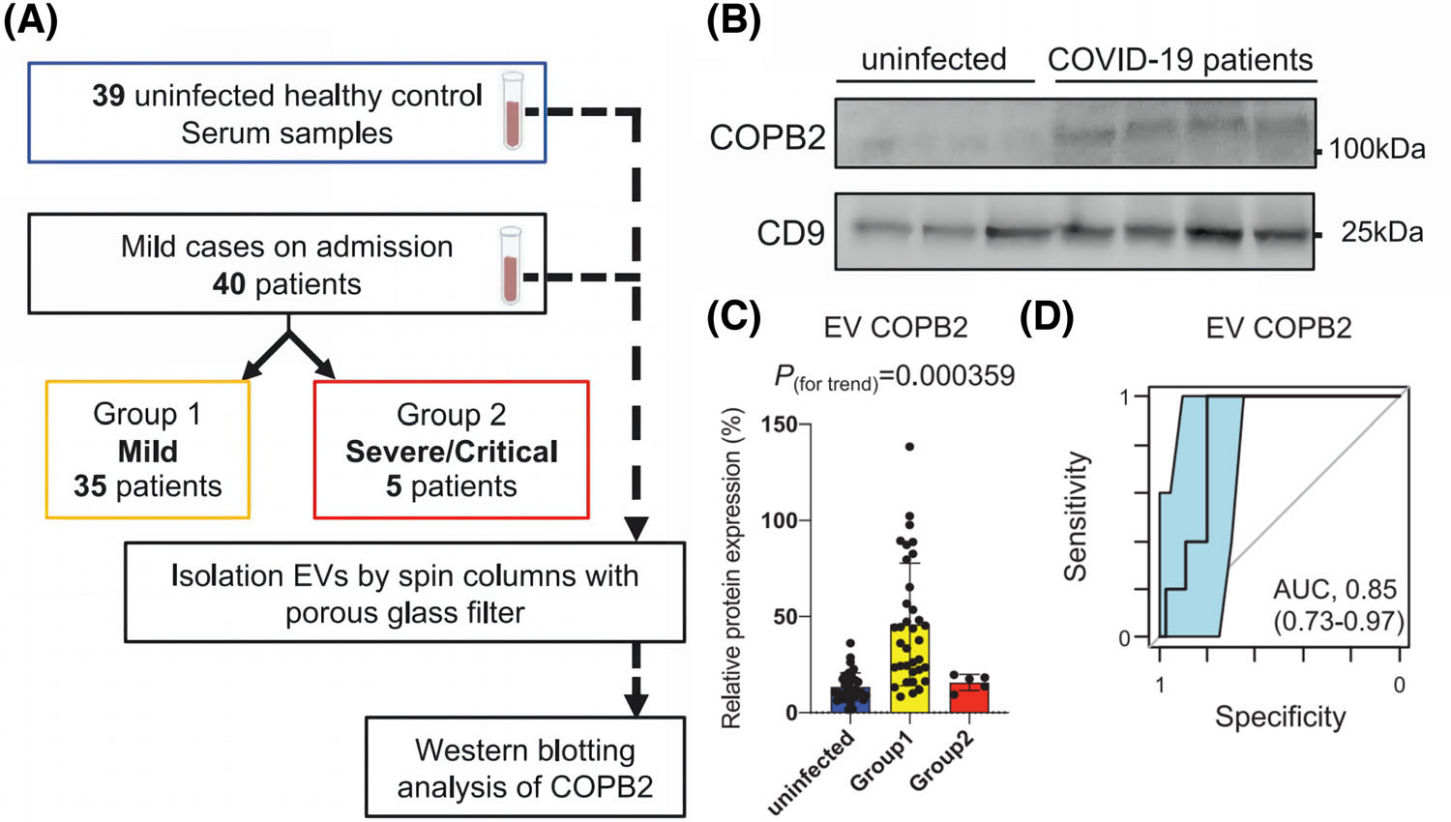
COPB2 in EVs is an early predictor of COVID‐19 severity. (A) Flowchart of Western blotting (WB) analysis of COPB2 in circulating EVs from 39 healthy individuals and 40 COVID‐19 patients (35 mildly ill (Group 1) and 5 severely/critically ill (Group 2)). (B) Representative images of WB of circulating EVs from healthy individuals and COVID‐19 patients. (C) Statistical analysis of the relative expression of COPB2 protein in circulating EVs from healthy individuals and Group 1 and Group 2 COVID‐19 patients. (D) Receiver operating characteristic curve (ROC) analysis and AUC values for Group 1 and Group 2. (Copyright (Fujita et al., 2021)) (COPB2, coatomer protein complex subunit beta 2).

In addition, coatomer protein complex subunit beta 2 (COPB2), a subunit of Golgi complex I (COPI), is a protein that plays a key role in SARS‐CoV replication (Thompson and Brown, 2012; Dey et al., 2022). Studies have shown that the expression of COPB2 in circulating EVs is substantially higher in patients with a mild infection symptom than in patients with a severe infection, suggesting that the expression of COPB2 in circulating EVs may be used as a predictor of disease regression (Fujita et al., 2021). (Figure 6) Mitchell et al. developed the ‘EV‐Catcher’ method for extremely selective purification and found that has‐miR‐146a ahashsa‐miR‐126‐3p, which are associated with anti‐inflammatory and vascular protection, were dramatically downregulated with disease severity (Mitchell et al., 2021). Thus, early detection of these brewing cytokines and timely blockage or replenishment would prevent the onset of more severe cytokine storm. Meanwhile, changes in circulating EVs may provide scientific guidance for identifying COVID‐19 patients who will be more serious.

#### 3.2.4 Alert the abnormal coagulation status

The morbidity and mortality of COVID‐19 are highly associated with thrombocytopenia, thrombosis, bleeding, and thrombotic sequelae. These sequelae raise alarm about the early detection of coagulation abnormalities and timely anticoagulation therapy (Rosenblum et al., 2021). Krishnamachary et al. added EVs from the plasma of COVID‐19 patients to lung endothelial cells and found that apoptosis was significantly promoted, reducing cell survival and leading to lung injury, implying that cardiovascular alterations in COVID‐19 patients may be associated with poor circulating EVs (Krishnamachary et al., 2021). The expression levels of tissue factor (TF), tissue plasminogen activator (t‐PA), von Willebrand factor (vWF), fibrinogen chain, and coagulation Factor V are all increased in poorly circulating EVs during the COVID‐19 hyperinflammation stage.

Alternatively, TF, the initiating factor of the coagulation process, is one of the most highly expressed markers for patients with severe COVID‐19 circulating EVs, suggesting that it may be a major cause of thrombosis (Krishnamachary et al., 2020). Rosell et al. found that SARS‐CoV‐2 infection induced a significant increase in TF activity on the surface of circulating EVs in vivo, and that this activity correlated with disease severity and may be associated with thrombosis in COVID‐19 patients (Rosell et al., 2021). Therefore, paying attention to changes in these indicators change may help prevent poor prognosis caused by abnormal coagulation. Balbi's team recently focused on tissue factor CD142, a receptor tissue factor that activates coagulation Factors X and IX. In this study, EVs were used as a prognostic factor for COVID‐19. They found that the exchange of EVs may be more active in the circulating blood of COVID‐19 patients, with a 2.7‐fold increase in concentration, compared with the control (Balbi et al., 2021). Furthermore, CD142 was significantly more highly expressed in severe COVID‐19 patients who underwent cannulation of suction and/or died, and by model analysis, they initially found that CD142 was 81.7% accurate as a prognostic predictor of COVID‐19, with high accuracy in excluding severe cases (Burrello et al., 2022). However, direct evidence is lacking to determine whether EVs with high CD142 expression directly contribute to venous thrombosis, and an increased sample size is needed to obtain more reliable results. Attention to changes in these indicators may help prevent poor prognosis caused by coagulation abnormalities.

#### 3.2.5 Portend the neurological dysfunction

Severe neurological inflammation and dysfunction often occur in patients who have recovered from COVID‐19 (Iadecola et al., 2020). Peluso et al. extracted EVs of neuronal and astrocytic origin from the plasma of COVID‐19 patients and found that both had the S1, RBD, and N protein of SARS‐CoV‐2. Among these COVID‐19 patients, EVs from patients with neurological symptoms abundantly expressed N proteins. In addition, mitochondria‐associated proteins, such as myosin VI (Myo6), subunit 6 of NADH‐ubiquinone oxidoreductase (respiratory chain complex I) (CI‐6) and CIII‐10, constituent proteins of the mitochondrial inner membrane electron transport chain, are dramatically reduced in EVs of COVID‐19 patients. These mitochondrial abnormalities reduce the efficiency of neural defence against the virus, implying that the mitochondria damaged by SARS‐CoV‐2 may be the key reason for psychiatric disorders (Peluso et al., 2022). Therefore, capturing aberrant mitochondria‐associated proteins in circulating EVs may trigger the onset of neurological dysfunction, and this discovery could lead to novel therapeutic targets.

#### 3.2.6 Provide hint for COVID‐19 therapy

Defensive effects against SARS‐CoV‐2 may include not only ACE2 on the surface of EVs but also EVs from surrounding MSCs and immune cells. In addition, researchers have also found many significant differences in circulating EVs from different populations. For example, compared to circulating EVs from young and healthy subjects, Wang et al. found that many miRNAs, including free miR‐7‐5p, miR‐24‐3p, miR‐145‐5p, and miR‐223‐3p, which directly inhibit the expression of S protein and the replication of SARS‐CoV‐2, were significantly reduced in circulating EVs from elderly and diabetic patients (Wang et al., 2021c). Many clusters of differentiation cytokines, such as CD147 and CD142, associated with platelet activation or coagulation, are dramatically changed in circulating EVs from patients with moderate to severe COVID‐19 (Balbi et al., 2021; Maugeri et al., 2022). The difference in miRNA and protein levels between healthy individuals and patients will inspire some potential hints for therapy.

### 3.3 Engineered EVs alleviated symptoms by calming the cytokine storm

#### 3.3.1 Engineered EVs

Overexpression of immune factor receptors on the surface of EVs is a promising strategy for neutralizing cytokines in the organisms. CD24 is an important checkpoint for controlling innate immune responses, which inhibit the NF‐κB signalling pathway and the production of cytokines, and CD24 knockout mice exhibit a lower inflammatory state after injury (Lee et al., 2016; Shapira et al., 2022). While CD24 inhibits immune activation induced by damage‐associated molecular patterns (DAMPs), this activation neither affects immune recognition of pathogen‐associated molecular patterns (PAMPs) nor interferes with virus clearance (Liu et al., 2009). Shapira et al. prepared EV agents with high CD24 expression and highlighted their safety in both animals and individuals. They found no organ damage or negative effects in mice, as well as no significant negative effects in individuals (Ib/IIa) for up to 443—575 days of follow‐up, indicating good biocompatibility. After 5 days of escalating doses (10^8^–10^10^ particles) of inhalation administration, 35 patients with severe COVID‐19 showed reduced release of inflammatory markers and cytokines/chemokines and at Day 7, more than 2/3 of patients with severe COVID‐19 displayed relief from symptoms (including coughing and polypnea) (NCT04747574; Shapira et al., 2022). Even without randomized designs, the study still brought some encouraging findings.

Furthermore, when the body is subjected to a cytokine storm, a large amount of reactive oxygen species (ROS) is produced, resulting in strong oxidative stress, causing damage to tissue cells and then leading to a more serious cytokine storm, so the control of oxidative stress is critical (Cecchini and Cecchini, 2020). The regulation of cytokines and antioxidants can effectively slow tissue damage. Ma et al. obtained EVs with antioxidant properties by co‐culturing polydopamine (pDA), which has free radical trapping ability, with HEK293T cells overexpressing hACE2. This strategy increased the ability of EVs to neutralize the SARS‐CoV‐2 S protein, and the release of pDA also slowed down the tissue and organ damage caused by oxidative stress (Ma et al., 2022).

#### 3.3.2 Biomimetic EVs

Interleukin‐6 (IL‐6), a proinflammatory cytokine, and GM‐CSF, a growth factor, are all the initiator of the cytokine storm (Fajgenbaum and June, 2020). Human leukaemia monocytic cells (THP‐1) contain numerous GM‐CSF receptors and IL‐6 receptors. Thus, NVs, fused with THP‐1 cells and HEK293T cells that were transfected with ACE2, not only act as decoys for neutralizing SARS‐CoV‐2, but also adsorb cytokines and prevent stronger immune responses. Twenty micrograms of NVs alone adsorbed approximately 160 pg IL‐6 and approximately 25 pg GM‐CSF, effectively reducing inflammatory responses and SARS‐CoV‐2 infection (Rao et al. 2020).

**FIGURE 7:**
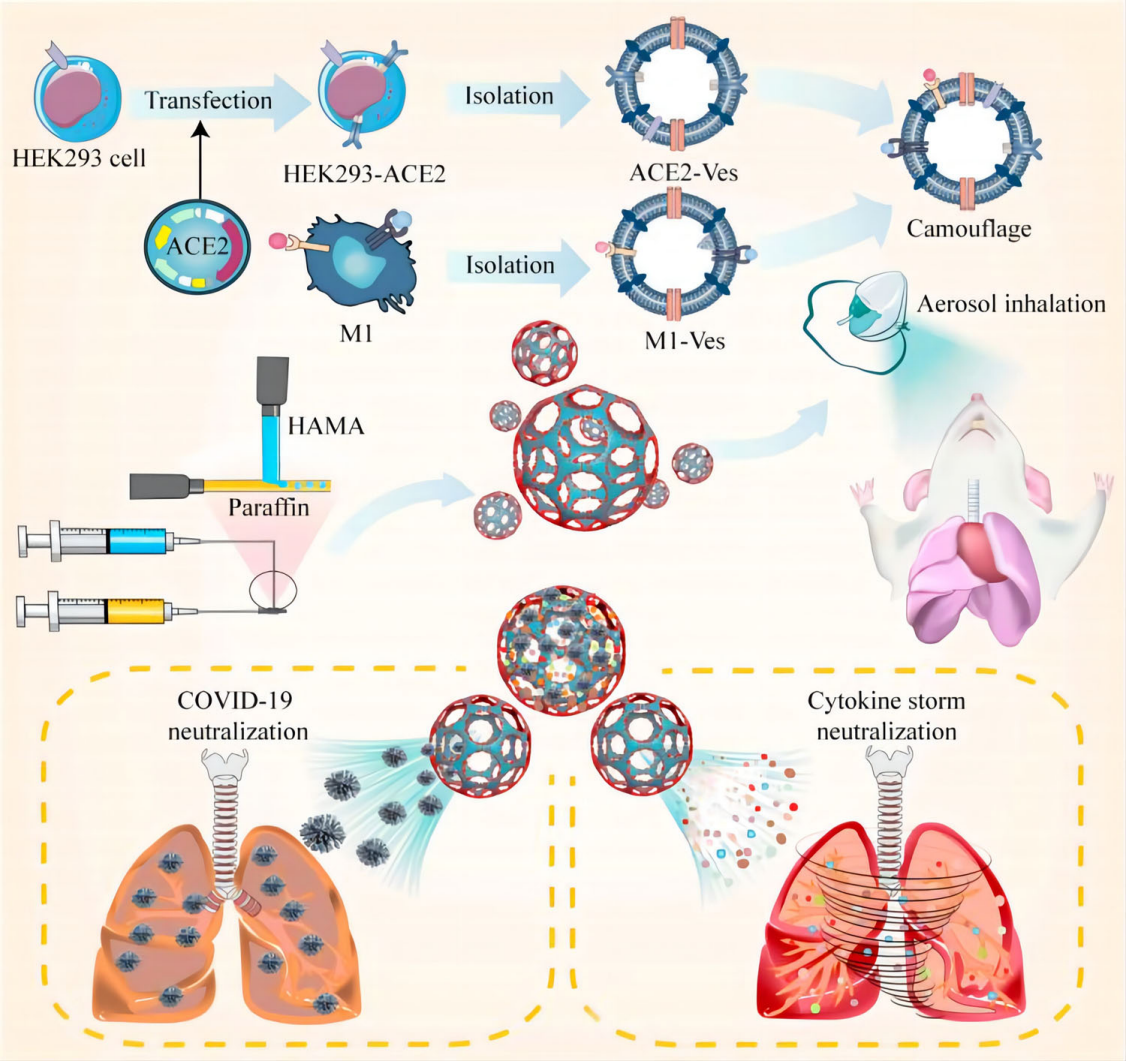
Schematic diagram of the inhaled microfluidic microsphere. The cell membranes of HEK293T cells transfected with ACE2 were fused with the cell membranes of M1 cells and modified with HAMA microspheres to form an inhaled microfluidic microsphere, which was used to treat mice by aerosol inhalation to neutralize both SARS‐CoV‐2 and cytokine storms. (Copyright (Wang et al., 2022b)) (HEK293T, human embryonic kidney 293; Ves, vesicles; M1, RAW264.7 macrophages that stimulated with LPS and TNF‐α; ACE2, angiotensin‐converting enzyme 2; HAMA, methacrylate hyaluronic acid).

Similarly, Wang et al. made an inhaled microfluidic microsphere by fusing the cell membranes of HEK293T cells transfected with ACE2 and RAW264.7 macrophages that stimulated with LPS and TNF‐α expressed high levels of receptors for IL‐6, IL‐1β, and TNF‐α, and then incubated them with methacrylate hyaluronic acid hydrogel microspheres in a 2:1 (w/w) ratio. (Figure 7) Compared to the blank control group, this strategy increased the antiviral efficiency from 9.28% to 91.33% and significantly alleviated the hyperinflammatory state of COVID‐19 by adsorbing inflammatory factors such as IL‐6, IL‐1β, and TNF‐α (Wang et al., 2022c). These findings further demonstrate that biomimetic EVs that via fused with cell membranes are a powerful ‘meet changes with constancy’ therapeutic strategy for promoting both SARS‐CoV‐2 and cytokine clearance. Furthermore, with the assistance of biomaterials, these benefits will be more long‐lasting and effective.

Plant‐derived exosome‐like nanoparticles (ELNs) also exemplify the anti‐inflammation and anti‐SARS‐CoV‐2 roles of biomimetic EVs because many plants have natural antiviral and anti‐inflammatory properties. The roles of plant ELNs in cross‐species communication, particularly in antiviral effects, have been widely demonstrated (Mu et al., 2014; Cai et al., 2018). miRNAs in plant ELNs, resembling miRNAs in MSC‐EVs, have been found to block SARS‐CoV‐2 replication and activity (Teng et al., 2021). For example, RNA‐dependent RNA polymerase (Nsp12) and RNA helicase (Nsp13) activate NF‐κB, triggering a cascade of inflammatory responses that lead to lung epithelial cell death and lung tissue damage (Wang et al., 2021b). Ginger‐derived ELNs contain high expression of aly‐miR396a‐5p, which inhibits Nsp12, and rlcv‐miR‐rL1‐28‐3p, which inhibits SARS‐CoV‐2 replication, providing a safe and effective strategy for preventing cytokine storms (Teng et al., 2021). In addition, Sundaram and his colleagues also identified 22 miRNAs in ginger and grapefruit ELNs that have potency for reducing SARS‐CoV‐2 replication, 11 of which showed absolute target specificities, such as miR‐5077, miR‐156a, and miR‐166 m. Surprisingly, many plant ELNs also had potential targeting ability; for example, ginger‐derived ELNs have been shown to be more likely to accumulate in lung tissue and impede the replication of SARS‐CoV‐2, but the mechanism is currently unclear (Kalarikkal and Sundaram, 2021).

## 4 RECOVERY: THE POTENTIAL OF EVS IN PROMOTING RECOVERY OF COVID‐19 PATIENTS

### 4.1 EVs as a therapeutic agent for COVID‐19 patient recovery

COVID‐19 patients improve and recover with the synergistic positive effect of drug treatment and organism defence. In a previous study, the role of cell‐derived EVs in tissue repair was systematically demonstrated and reviewed (Brennan et al., 2020; Kim et al., 2022b). Although numerous researchers from the International Society for Cell and Gene Therapy (ISCT) and the International Society for Extracellular Vesicles (ISEV) claimed that biosafety issues should be considered in the clinical application of EVs, the mechanisms of potential therapeutics may still be unknown in March 2020 (Börger et al., 2020). Clinical trials have shown some positive results indicating that the potential of EVs can help patients recover from COVID‐19 (Sengupta et al., 2020a; Zhu et al., 2022). Based on these trials, we will focus on the potential of EVs in COVID‐19 recovery in this section.

A prospective study examined the safety and efficacy of bone marrow mesenchymal stem cell exosomes (MSC‐exos) in 24 patients with moderate and severe COVID‐19 pneumonia (Sengupta et al., 2020a). At approximately 5.6 days after intravenous injection, 71% of patients recovered, 13% of patients remained critically ill although their conditions are stable, and 16% of patients expired for reasons unrelated to the treatment. More importantly, no significant side effects were observed in almost all patients within 72 h of the injection. After 14 days, the oxygenation of the patient improved and recovered. Lim et al. worried that this study contains many things that cannot be explained and that biosafety should be considered (Lim et al., 2020). Lim et al. further discussed manufacturing specifications of the products, biological activity (of which 40% are proteins that are related primarily to immune regulation), dosage (15 ml, with a concentration of 40 million cells/ml), and patient‐specific vital signs after treatment (continuous monitoring of cardiac and pulse oximetry (SpO_2_) after surgery) (Sengupta et al., 2020b). Insufficient clinical samples are also one of the disadvantages. This study suggested that the injection of MSC‐exos may be a promising therapeutic method for the treatment of patients with COVID‐19. On the other hand, other studies also found that MSC‐exos can improve the permeability of the alveolar epithelium and reduce the severity of lung injury by increasing the number of anti‐inflammatory signalling molecules (Abraham and Krasnodembskaya, 2020; Dabrowska et al., 2020).

In another clinical pilot study published recently by Zhu et al., seven severe COVID‐19 patients were recruited to receive aerosolized clinical‐grade exosomes derived from human adipose‐derived mesenchymal stem cells (MSCs) for five continuous days. Lung computed tomography (CT) showed that all patients revealed varying degrees of regressive lesions, and 57% of patients responded well to the treatment. No significant side effects were observed during the process of aerosol inhalation. However, in this study, both pharmacological therapy and netted nebulization inhalation of MSC‐exos were used at the same time, and in addition to insufficient samples, it is unclear how much real effectiveness MSC‐exos spray will make (Zhu et al., 2022) (NCT04276987). All these findings show that treatment with stem cell‐derived EVs could play roles in anti‐SARS‐CoV‐2 and alleviating COVID‐19‐related inflammatory responses. However, many other details need to be further examined, such as exosome dosage, administration interval, the impact of administration manner on therapeutic effect, actual exosome bioavailability, mechanism, and biosafety assessment. Furthermore, the indication of these strategies should also be considered.

In addition, severely ill COVID‐19 patients have many sequelae after recovery, and pulmonary fibrosis is the most common feature. Antifibrotic therapies, such as tadalafil and pirfenidone, may play a role in attenuating the profibrotic pathway of SARS‐CoV‐2 infection, but these therapies are not economically sustainable and have serious adverse effects. EVs may provide a new therapeutic approach for the treatment of COVID‐19 pulmonary fibrosis through their anti‐inflammatory and antifibrotic factors. The destruction of alveolar structure, abnormal fibroblast proliferation and inappropriate extracellular matrix (ECM) accumulation are the main causes of pulmonary fibrosis, inhalation of lung spheroid cell exosomes (LSC‐Exos) can partially correct these abnormalities. Furthermore, LSC‐exos showed better efficacy than MSC‐exos in certain indices. Notably, existing studies have focused on EV recovery effects in COVID‐19 (Sengupta et al. 2020a; Zhu et al., 2022); however, no studies have confirmed the role of EVs in the process of treating other COVID‐19 sequelae, such as fatigue, pain, and difficulty concentrating. Studies on the direct involvement of EVs in the repair of COVID‐19‐associated lesions in vivo are still lacking, which means further breakthroughs in tracer systems of EVs should be made.

### 4.2 Engineered EVs targeted to damaged tissue

**FIGURE 8:**
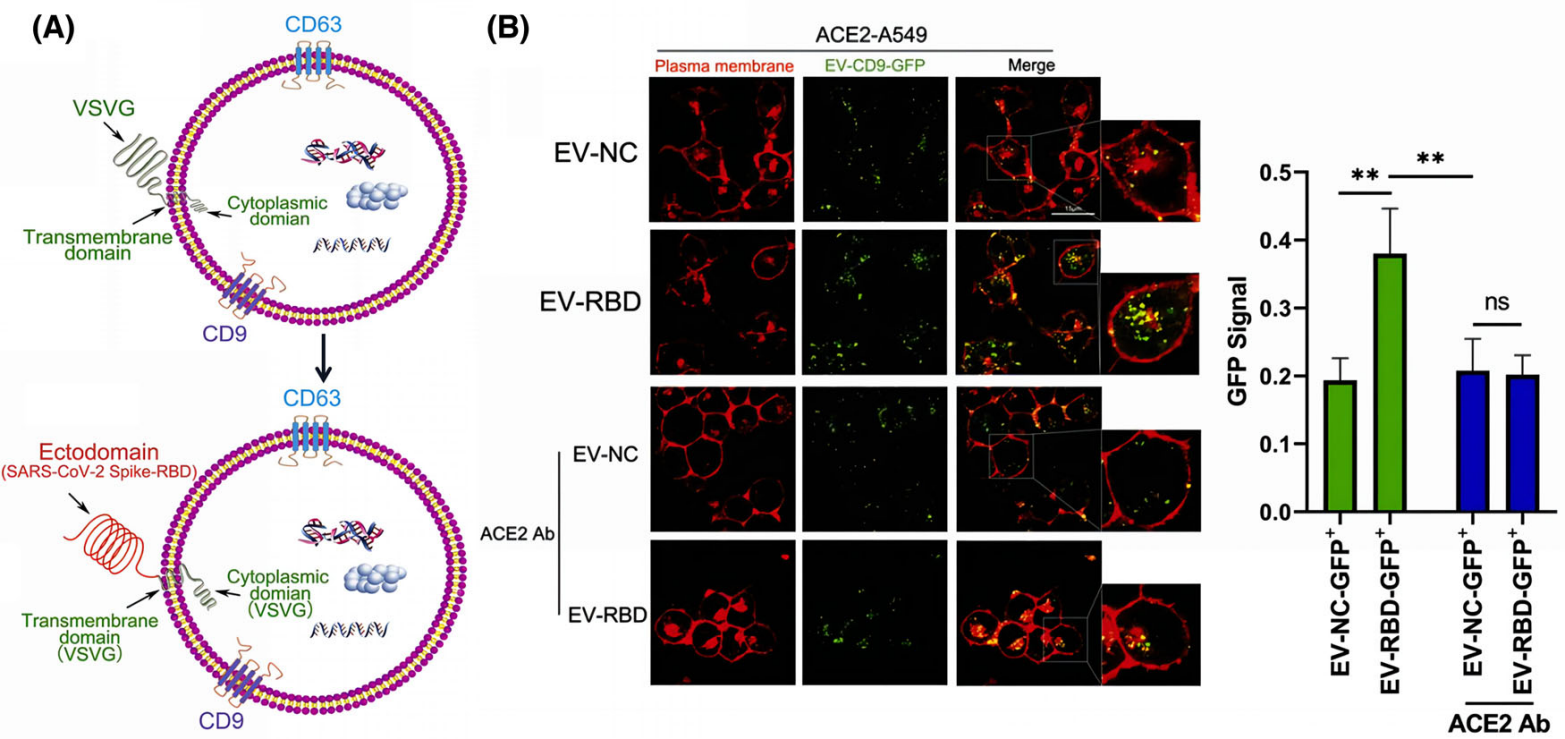
Strategy to promote the targeting ability of EVs to cells with high ACE2 abundance using VSVG as a SARS‐CoV‐2 spike‐RBD scaffold. (A) The VSVG pseudotyped virus was used to load the SARS‐CoV‐2‐RBD protein onto EV membranes. (B) Representative fluorescence images and statistical analysis plots with/without ACE2 Ab pretreatment and then with EV‐NC or EV‐RBD coincubated with cells for 6 h. (**P < 0.01) (Copyright (Fu and Xiong, 2021)) (ACE2, angiotensin‐converting enzyme 2; EV, extracellular vesicles; RBD, receptor‐binding structural domain; VSVG, vesicular stomatitis virus‐G glycoprotein).

Specific targeting and repair of damaged tissue is a very interesting topic. Because there is a high affinity between the receptor binding domain (RBD) of SARS‐CoV‐2 and human ACE2, cells and tissues with high ACE2 expression become the major targets of SARS‐CoV‐2 colonization and destruction, which means they need to be repaired after SARS‐CoV‐2 infection (Tai et al., 2020). Fu et al. adopted a ‘meet change with constancy’ strategy by using vesicular stomatitis virus‐G glycoprotein (VSVG) to load the SARS‐CoV‐2‐RBD protein onto EV membranes, and these EVs probably target ACE2 receptor‐rich cells and tissues (Fu and Xiong, 2021). (Figure 8) In addition, Ibrahim et al. found that transfection of lentiviral β‐catenin and the transcription factor GATA‐4 into fibroblasts resulted in an efficient yield of EVs, which were named ASTEX. Compared to fibroblast‐derived EVs, ASTEX which contains more transcription factors, abundant piRNAs and miRNAs, including miR‐183 and miR‐182, and fewer complement activators, can exert anti‐inflammatory and antifibrotic effects. Therefore, ASTEX demonstrated good repair effects by reducing bleomycin‐induced pulmonary fibrosis and alleviating lung inflammation, and also greatly improved the survival rate of SARS‐CoV‐2 infected cells (Ibrahim et al., 2021). COVID‐19 recovery should not only focus on ACE2‐expressing cells, but also be concerned about the rampant cytokine storm in the body. Cytokine storms indiscriminately damage tissues and organs (Ye et al., 2020), in which serious sequelae have been observed. Furthermore, the sequelae of COVID‐19 patients have not yet been fully discovered.

## 5 IMMUNITY: EVS PARTICIPATE IN SARS‐COV‐2 IMMUNITY

### 5.1 EVs assist in and witness acquired SARS‐CoV‐2 immunity

After SARS‐CoV‐2 infection or vaccination, circulating EVs contribute to the production of antibodies and protective immunity, including cellular and humoral immunity (Miyashita et al., 2022; Pesce et al., 2022; Tsai et al., 2021). Bansal et al. found that 14 days after the first vaccination, circulating EVs began to transport the S protein of SARS‐CoV‐2, and antibodies were found in circulating EVs 14 days after the second immunization. There was a 12‐fold increase in antibody levels compared to 14 days after the first vaccination. Four months after the second vaccination, the spike protein and antibody levels decreased (Bansal et al., 2021). However, detailed and long‐term tracking information of antibodies and S protein in EVs was lacking in this study. Further experiments, such as direct inhibition of EVs, should be designed to verify whether EVs play an irreplaceable role in acquired immunity to COVID‐19. EV‐associated antibodies may be more stable and relatively easier to preserve than soluble and unbound antibodies. The EV‐associated antibodies are less susceptible to degradation, and the enriched antibodies may be displayed on the surface of exosomes and provide a more powerful capacity to fight against viruses (Ogawa et al., 2021; Jeyaram and Jay, 2017). If the source of EVs can be traced with the development of the technologies, greater significance will be attached to the test results. Of course, the tedious isolation steps before decrypting information from EVs should be considered.

Induction of specific CD8^+^ T‐cells mediated immunity to SARS‐CoV‐2 is also critical for long‐term immune memory and disease prognosis (Rydyznski Moderbacher et al., 2020; Peng et al., 2020). Studies have shown the presence of SARS‐CoV‐2 spike‐derived fragments on the surface of EVs in the circulating blood of mildly ill COVID‐19 patients. These EVs that were possibly captured by antigen presenting cells (APCs), can act as a source of antigen presentation to activate T cells (Pesce et al., 2022), which means activation of cellular immunity. In addition, some results demonstrated that using EVs as a delivery vehicle can activate cellular immunity. Nef^mut^, a biologically inactive human immunodeficiency virus (HIV) type 1 Nef protein, has a very high efficiency of incorporation into EVs by fusing foreign peptides at the C‐terminus (Lattanzi and Federico, 2012). Federico's team has performed a series of studies using this strategy, they fused S1 and S2, N, and M of the S protein of SARS‐CoV‐2 to the C‐terminus of Nef^mut^ and found that this fusion protein efficiently bound to EVs. Strong specific CD8+ T‐cell immunity was elicited after a single intramuscular injection, and the immunity was present in the pulmonary airways (Ferrantelli et al., 2021). They found that N‐specific CD8^+^ T lymphocytes were 100% resistant to lethal amounts of SARS‐CoV‐2 infection and could induce strong N‐specific CD8^+^ T cytotoxic lymphocyte (CTL) immune responses, but S1‐specific CD8^+^ T lymphocytes were less effective (Ferrantelli et al., 2022; Manfredi et al., 2022). The possible effects of this vaccine also need to be evaluated over a longer time period.

### 5.2 Engineered EVs as more efficient SARS‐CoV‐2 vaccine carriers

Classic molecular techniques, such as inactivated or attenuated viruses, single peptides, or viral vectors, are currently being used for vaccine development (van Riel and de Wit, 2020). However, there are several problems, including the possibility of virus re‐emergence and failure to provide long‐term protection. Additionally, newly developed RNA vaccines have demonstrated good protection rates, quick development cycles, and simple production, but they are subject to strict preservation conditions, have a limited half‐life, and rarely painful or allergic reactions. (Brisse et al., 2020; Crommelin et al., 2021; Holm and Poland, 2021; Kelso, 2021; Skowronski and De Serres, 2021). Many companies have developed vaccines after decoding the full genetic fragment of SARS‐CoV‐2, but the primary immune antibodies of existing vaccines target only a portion of the full‐length S protein, necessitating the use of boosters in the face of highly variable S protein strains such as Omicron (Chen et al., 2021b; Khan et al., 2022).

To address these shortcomings of existing COVID‐19 pneumonia vaccines, a modified approach is needed to develop safe and effective vaccines that provide long‐term immunity. EVs may open a new path for the development of effective novel COVID‐19 pneumonia vaccines (Jiang et al., 2022; Sabanovic et al., 2021). EVs have shown some intuitive advantages as carriers for pathogenic vaccines: (1) greater potency; (2) induction of higher specific CD8^+^ T‐cell and B‐cell immunity; (3) crossing of the placental barrier and generation of neonatal immunity; (4) less toxicity; (5) expanded molecular distribution by reaching distal organs through body fluids; (6) potential for large effects without immune adjuvants; (7) higher encapsulation efficiency and (8) provision of a stable protective environment for RNA and proteins. (Santos and Almeida, 2021; Tsai et al., 2021)

#### 5.2.1 Engineered EVs

Several technology companies have reported two ways to develop EV‐based COVID‐19 vaccines: one is to use EVs as a carriers and load the major structural protein fragment of SARS‐CoV‐2 directly into EVs; the other is to transfect source cells with fragment information from SARS‐CoV‐2 and produce EVs that carry the relevant antigens and proteins (Sabanovic et al., 2021; Yoo et al., 2022). Tsai et al. created the LSNME/S^W1^ vaccine, which uses mRNA containing L, S, N, M, and E proteins and full‐length functionalized S proteins loaded into purified EVs and vaccinated on Days 1, 21, and 42. They found that after receiving this vaccine, the organisms produced antibodies against the N and S proteins, which remained stable until week 7, albeit at low concentrations. This is because the main goal of the vaccine is to activate protein‐mediated cellular immunity, and they found that the addition of S and N proteins to the vaccine successfully activated reactive CD4^+^ and CD8^+^ T cells (Tsai et al., 2020). Ciloa created the CoVEVaX vaccine, which is based on their unique CilPP guide peptide technology, which allows cells to load grafted fragments onto EVs. The SARS‐CoV‐2 spike protein is attached to the C‐terminus of the CilPP peptide (DNA^S‐EVs^), which contains the tip of S1 and S2 proteins in their normal configuration, as well as the usually overlooked metabotropic glutamate receptor 5 and transmembrane proteins. They also created S‐EVs by infecting cells directly with SARS‐CoV‐2 and S‐Trim by transfecting cells with the DNA^S‐Trim^ plasmid. They tested a combination of these three designs and discovered that two doses of DNA^S‐EVs^ (initial vaccination) plus one dosage of S‐Trim or S‐EVs (booster) provided significant cellular immunity, with no further viral components or immune adjuvants needed (Polak et al., 2020). Alle Biotechnology and Pharmaceuticals, Codiak BioSciences, and Versatope Therapeutic also found that EV‐based COVID‐19 vaccines are easier to store than existing vaccines and that EVs can carry more mRNA and protein than single adenovirus vaccines, resulting in stronger effects (Sabanovic et al., 2021).

In addition, recent studies have shown that nasal inhalation of vaccines can mimic the entry of SARS‐CoV‐2 into the respiratory system and exert mucosal immunity in key areas compared to intramuscular injection (Huang et al., 2022). Afkhami et al. found that their nasal inhalation adenovirus vaccine produced the same immune impact at a 1% intramuscular dose. This finding implied that an equivalent amount of vaccine administered via nasal inhalation would benefit nearly 100 people, saving money and materials (Afkhami et al., 2022). Thus, EVs combined with nasal nebulized administration may be able to achieve better immune effects.

Several teams have attempted to develop new inhaled vaccines based on EVs. Wang et al developed a novel inhaled vaccine by affixing the RBD region to the surface of exosomes derived from LSCs (Wang et al., 2022c). This vaccine has the following benefits: (1) Compared to liposomes, exosomes are more retained in the lung and enter the fine bronchi, with higher bioavailability, (2) Amended exosomes remain effectively active after 6 months at 40°C ± 2°C/75% ± 5% relative humidity, suggesting that the product has good stability against degradation. Inhaled vaccines overcame the problem that existing vaccines must be transported in the cold chain, reduced transportation costs and facilitated vaccine distribution and dispensing, (3) The RBD attached to the surface of exosomes was more readily taken up by antigen‐presenting cells, and the SARS‐CoV‐2 mimics were more rapidly cleared from hamsters by spray administration than by intravenous administration and (4) Compared to intramuscular administration with aluminium adjuvant, spray administration produced lower RBD‐specific Immunoglobulin G (IgG) in body fluids but 10‐100‐fold higher specific sIgA antibody titres, suggesting activation of mucosal immunity. Additionally, the spray activated CD4+ and CD8+ T‐cells to produce IFN‐γ, which strongly induced of a systemic T‐cell response. Notably, Popowski et al. used lung‐derived EVs as carriers loaded with mRNA or protein cargo, EVs were lyophilized and stored at room temperature for 28 days, and the cargo in EVs remained effectively active, with less than 2.4% of the total cargo (pg/ml) leaking (Popowski et al., 2022). They further loaded the S protein of SARS‐CoV‐2 into lung EVs, monkeys and mice and produced more protective antibodies IgG and sIgA, which can clear the pseudovirus more effectively after inhaling dried EV powder compared to liposomes as carriers.

#### 5.2.2 Biomimetic EVs

**FIGURE 9:**
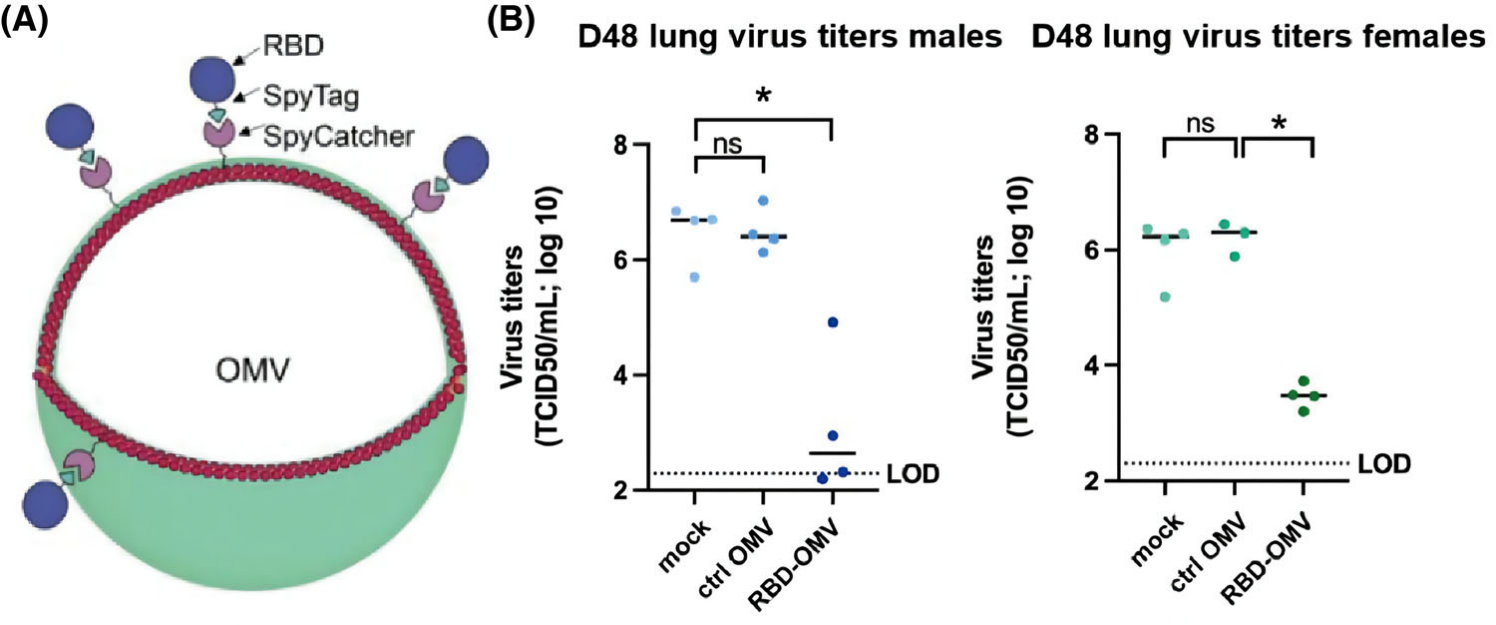
The OMV‐RBD vaccine prevents SARS‐CoV‐2. (A) Strategy diagram of the OMV‐RBD vaccine. (B) The viral titres in the lungs of male and female hamsters at Day 48 via nasal inhalation of the OMV‐RBD vaccine. (*P < 0.05) (Copyright (Jiang et al., 2022)) (OMV, outer membrane vesicles; RBD, Receptor‐binding domain).

Based on the consideration of the low yield of EVs, Jiang et al. used engineered bacteria that can replicate rapidly, have low virulence, and produce large numbers of outer membrane vesicles (OMVs) to develop the vaccines. They decorated the RBD on the surface of the OMV to develop the OMV‐RBD vaccine and administered it by nasal inhalation, which showed no significant changes in hamster body weight and temperature. (Figure 9A) After 42 days of intranasal administration, anti‐spike‐RBD specific IgGs were found in both plasma and bronchoalveolar lavage, indicating effective humoral and mucosal immunity. Furthermore, the RBD‐OMV group had much lower viral titres (100‐ to 1000‐fold) than the control group (OMV group), and viral pathogenicity to the organism was significantly reduced (Figure 9B). This OMV‐RBD vaccine, which targets the RBD region of the wild‐type SARS‐CoV‐2‐S protein, is still effective against Delta mutant strains (Jiang et al., 2022). The above vaccination has not been tested in the extremely infectious Omicron subvariants that are now in the pandemic. However, these groundbreaking studies will provide new insights for vaccine development.

Because SARS‐CoV‐2 enters the body mainly through the respiratory tract, exosome‐based nasal inhalation administration vaccines seem to have many advantages, especially strong mucosal immunity and biocompatibility. Furthermore, through nasal administration combined with intramuscular injection achieves stronger immunity is also a topic worth exploring. Whether the consistency and stability of the engineered approach can be guaranteed and whether exosome‐based vaccines can overcome the economic benefits in terms of production costs remain sensitive points. The results could be more instructive if currently available, widely used vaccines were used as standard controls. More notably, none of the vaccines have been tested in preclinical trials.

## 6 CHALLENGES: EVS CLINICALLY FIGHT AGAINST SARS‐COV‐2

**FIGURE 10:**
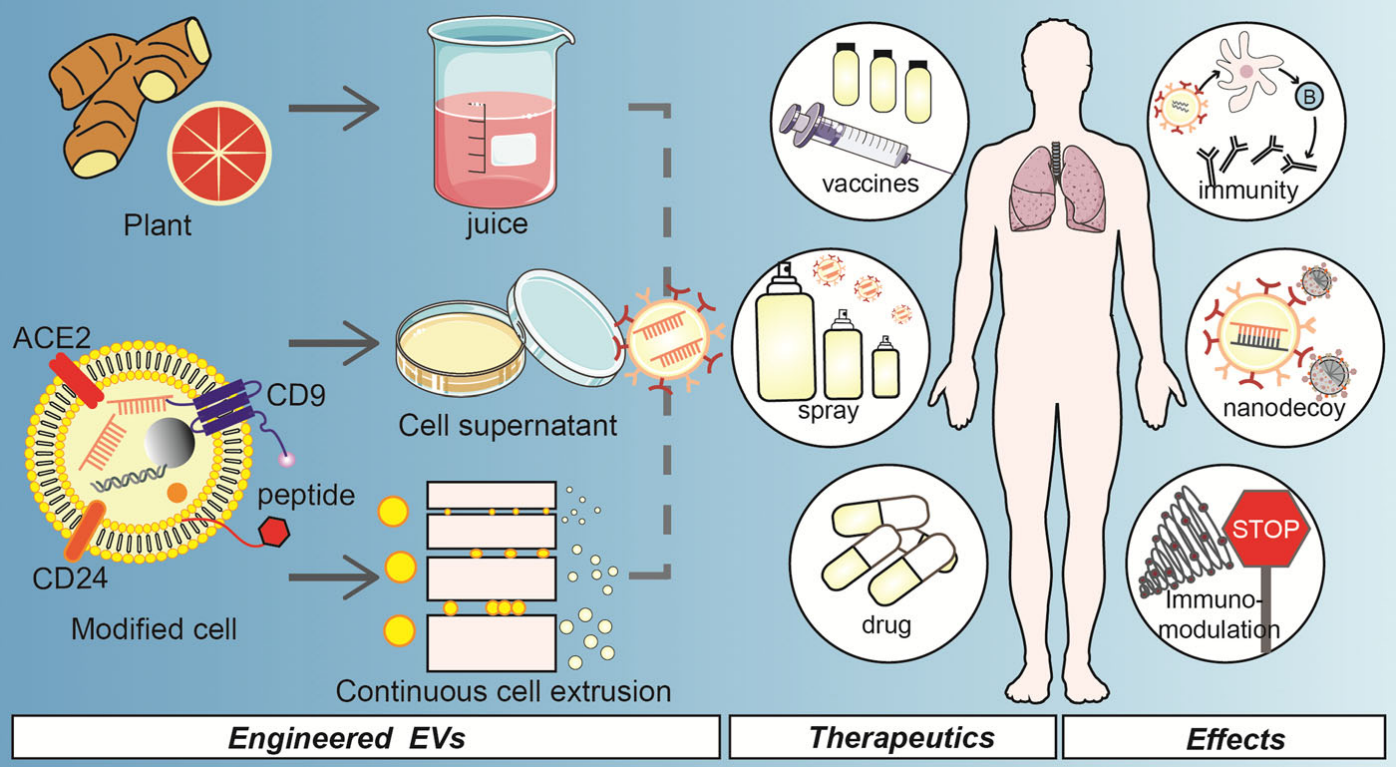
The therapeutic effects of engineered and biomimetic EVs in COVID‐19. (ACE2, angiotensin‐converting enzyme; CD, cluster of differentiation; EVs, extracellular Vesicles).

The above four processes of EVs against SARS‐CoV‐2 provide a range of diagnostic, therapeutic and preventive strategies to overcome COVID‐19, especially the amplification of therapeutic effects by engineered and biomimetic EVs. (Figure 10) (Table 1) However, it is critical to recognize that EV research is currently confronted with a number of issues that must be addressed, such as how to overcome the low yield of EVs mentioned above, and various efforts have been made by researchers, including gene editing and finding alternatives. In this section, we will discuss the key challenges currently facing EVs as anti‐SARS‐CoV‐2 therapies in the clinic, focusing on the selection of EV sources, possible biosafety issues, and how to maximize the economic effects.

### 6.1 Lack of detailed study of mechanism

EVs play a crucial role in the physiological and pathological processes of the organism (Thakur et al., 2021; Yates et al., 2022a, 2022b), such as their roles in defence, antagonism, repair, and immunity in the pathogenic phase of SARS‐CoV‐2, but more detailed mechanistic studies are lacking. On the one hand, numerous key details have been overlooked in previous research. For example, SARS‐CoV‐2 RNA has been found in circulating EVs from COVID‐19 patients, but the assessment of their pathogenicity and activity has not been published (Ning et al., 2021). On the other hand, the current study relied on EVs from cells cultured in vitro or circulating EVs, and direct visualization studies in vivo are still scarce. Additional study will provide new inspiration for treatment if the interaction process between EVs and SARS‐CoV‐2 can be traced using a more mature transgenic animal model with EV‐special green fluorescent protein (GFP).

### 6.2 Source selection for EVs

1. Abundance of cell surface receptors. BMSCs, LSCs, HEK293T cells, Vero‐6 cells, and primary peritoneal M2 macrophages were employed as tools to produce EVs for treating COVID‐19 (Sengupta et al. 2020a; Li et al., 2021; Xie et al., 2021; Wang et al., 2022b; El‐Shennawy et al., 2022). (Table 1) In terms of the abundance of ACE2 expression, the current naturally unmodified cells with higher ACE2 abundance are LSC‐NVs, which are 10‐fold more abundant than NVs from HEK293T cells (Li et al., 2021). However, cell lines may be a superior alternative for transfection of cells with higher ACE2 expression since they are easier to transfect and develop quickly (Abaandou et al., 2021).
2. Considerations for yield. There has been some systematic review on how to increase the yield of EVs (Hao et al., 2021). The challenge we need to highlight here is the urgency to consider that current strategies not only increase the yield of EVs, but also may affect the contents of EVs. For example, 3D culture has been shown to improve the ability of cells to produce EVs, and their secretomes have been shown to have a greater effect on improving survival in mice with acute lung injury caused by LPS than that of 2D culture (Thippabhotla et al., 2019; Kudinov et al., 2022).

### 6.3 Biosafety of therapeutic agents

One of the most significant factors before entering the clinic is the biosafety of diverse modified EVs, which includes short‐ and long‐term harmful effects on the organism, including cytotoxicity, organ damage, and inflammatory reactions.

1. Elucidation of clearance mechanisms. Although EVs have been shown to be effective as nanodecoys for SARS‐CoV‐2, the specific clearance procedure of EVs adsorbed to SARS‐CoV‐2 is unknown, and both mucociliary cilia and macrophage transport are considered feasible clearance routes (Fu and Xiong, 2021; Zhang et al., 2021a). There is currently a bottleneck in monitoring EVs in the body, which necessitates the development of more effective tracer imaging techniques and methodologies for detecting EV behaviour in cells and tissues (van Niel et al., 2022).
2. Monitoring the EV source. Although most of the EVs mentioned above are derived from animal cells, some reported risks related to the safety of EVs should also be considered. EVs derived from adipose stem cells have a higher risk of thrombosis than EVs derived from bone marrow mesenchymal stem cells (Chance et al., 2019; Silachev et al., 2019). Furthermore, HEK293T cells are the most common source for extracting EVs in this field, and the potential adverse effects of cell quality control cannot be overlooked. Studies have shown that HEK293T cells with more than 65 passages will generate tumour tissue after continuous injection into mice for 2 weeks, whereas cells with fewer than 52 passages have a higher safety profile (Shen et al., 2008).
3. The heterogeneity of EVs. Sequencing tests such as proteomics and lipidomics have confirmed the complexity of EVs, including affluent content and surface composition, but it is unknown whether these complex components would have deleterious impacts, which is one of the primary barriers to the clinical application of EVs. Fortunately, biomimetic EVs, including cell membrane nanovesicles, have emerged in recent years in response to the heterogeneity challenge of EVs (Szatmári et al., 2019; Chen et al., 2021a).

### 6.4 Maximization of economic benefits

In terms of economic effects, it is vital to evaluate how to maximize therapeutic effects in addition to the lowest feasible cost in the production line.

1. As a diagnostic tool. Circulating EVs may be a promising tool for detecting earlier, monitoring disease regression, and screening susceptible populations. However, the main difficulty is how to translate these findings in a cost‐effective manner, overcoming costly charges such as kit development and EV separation. Furthermore, sensitivity and specificity must be ensured. With advances in nanoflow cytometry technology, tracing the source of circulating EVs has become possible, such as accurately counting and sorting neutrophil‐derived EVs from circulating EVs, which is important for precise diagnosis (Bonifay et al., 2022).
2. Reproducibility and cost‐effectiveness of modification methods. Gene editing approaches, such as transfection, are the principal strategies for modified EVs discussed in this review. However, this gene editing frequently necessitates the use of pricey reagents, is inefficient, and does not guarantee reproducibility (Chong et al., 2021). It is also important to investigate whether transfection causes changes in the expression of other composites in the cell (Kim and Eberwine, 2010). Alternative solutions can be found in a variety of physical and chemical approaches. For example, Lathwal et al. have discovered ways to directly graft polymers onto the surface of exosomes using atom phototransfer radical polymerization (ATRP), which are highly controlled, cost‐effective, and can improve ex vivo stability, but there is still a long way to go for further application (Lathwal et al., 2021).
3. Storage approaches. In addition to addressing the impact of various temperatures on the stability of EVs, there is a need to think about how to maximize therapeutic efficacy retention (Lőrincz et al., 2014). Different cryoprotectants protect EVs in different ways; the most frequently used are sucrose, alginate, and mannitol, which can be used to store EVs for several years (Kusuma et al., 2018). Liu's group reported that nano‐EVs sprays protected by sucrose and stored at 4°C for 1 month had the best ability to neutralize SARS‐CoV‐2 (Zhang et al., 2021a). However, this does not constitute standard guidance. In addition, there are few systematic investigations on the thermal stability of EVs, and only a few studies have demonstrated that exosomes remain stable at 42°C for 8 h (Dang et al., 2017), and their protein quantity is dramatically reduced at 60°C (Cheng et al., 2019).
4. Mode of administration. Because SARS‐CoV‐2 enters the body mostly through the respiratory system, nasal nebulized inhalation is quickly becoming the most effective method of treatment (Zhang et al., 2021a). Several clinical trials are currently underway that also consider dosing intervals and doses, including interval or continuous dosing, fixed dose or dose escalation continuous dosing. In a study of twice‐daily dosing for 10 consecutive days, EVs did not improve indications such as time to discharge, although C‐reactive protein was significantly lower in the treatment group on Day 11 than in the placebo group (NCT04491240). Engineered EVs were also found to decrease mortality and lessen the body's immunological response of the body when given once daily for 5 days in another study (NCT04747574). These completed clinical studies appear to show that dose versus interval has a major impact on the efficacy of a drug.

### 6.5 Urgent clinical trials in the future

1. Challenges in animal models. The severity and complexity of the cytokine storm caused by COVID‐19 in humans cannot be fully replicated in animal models. New methods of tracking drugs in vivo at the animal level still need to be developed.

2. Increasing the sample size. Whether the information of circulating EVs can act as a clinical indication in prognosis and sequelae deserves further exploration. Assessing the sensitivity and specificity of this strategy also requires an increase in sample size, which also challenges the extractive methods of EVs. To evaluate the defence, antagonism, repair, and immune effects provided by EVs, further randomized designs, comprehensive assessments, and extended observation times are also needed to increase the sample size. In addition, timely sharing and analysis of data is expected to improve the accuracy of information from EVs.

## 7 CONCLUSIONS AND PERSPECTIVES

In conclusion, due to their natural mission, EVs have the honourable task of defending against infection, and preventing SARS‐CoV‐2 replication and dissemination, but when the organism falls, they may be forced to become prisoners or accomplices of SARS‐CoV‐2, contributing to the cytokine storms and distant infections. A new approach for battling SARS‐CoV‐2 is the production of diverse engineered and biomimetic EVs to boost antiviral effects. A new diagnostic strategy for the early diagnosis and prevention of unfavourable regression may be achieved by capturing and decoding intelligence in circulating EVs. EVs from the laboratory may also be a promising strategy for promoting the recovery of COVID‐19 patients, assisting in SARS‐CoV‐2‐related immunity. Most excitingly, ACE2, cytokine receptors, and internal miRNAs on EVs may offer a variety of ‘meet changes with constancy’ strategies for combating the ever‐mutating SARS‐CoV‐2.

For translational research in this field, biosafety, EV source, and economic benefit are currently factors to consider. Although there is still a long way to go, research has opened up new horizons in the prevention, diagnosis, and treatment of COVID‐19, and clinical studies have already begun. There are at least 422 clinical trials (https://clinicaltrials.gov/) on EVs underway as of November 2022, and 24 of those trials are seeking to employ EV to treat COVID‐19, with many more patents or products in the works. In an era of increasingly robust EVs research, this tiny vesicle could be a hope for overcoming SARS‐CoV‐2.

## AUTHOR CONTRIBUTIONS

Conceptualization, Xiaohang Chen, Huifei Li, Pengcheng Han, and Xing Wang; Funding acquisition, Xing Wang; Visualization, Xiaohang Chen, and Xing Wang; Writing – original draft, Xiaohang Chen; Writing – review & editing, Xiaohang Chen, Huifei Li, Haoyue Song, Jie Wang, Xiaoxuan Zhang, Pengcheng Han and Xing Wang. Supervision, Xing Wang. Resource, Xing Wang. All authors have read and agreed to the published version of the manuscript.

## CONFLICTS OF INTEREST

There are no conflicts to declare.
